# Comparing the model-simulated global warming signal to observations using empirical estimates of unforced noise

**DOI:** 10.1038/srep09957

**Published:** 2015-04-21

**Authors:** Patrick T. Brown, Wenhong Li, Eugene C. Cordero, Steven A. Mauget

**Affiliations:** 1 Earth and Ocean Sciences, Nicholas School of the Environment, Duke University, Durham, NC 27708; 2 Department of Meteorology and Climate Science, San José State University, San José, CA 95192; 3 Agricultural Research Service, United States Department of Agriculture, Lubbock, TX 79415-0000

## Abstract

The comparison of observed global mean surface air temperature (GMT) change to the mean change simulated by climate models has received much public and scientific attention. For a given global warming signal produced by a climate model ensemble, there exists an envelope of GMT values representing the range of possible unforced states of the climate system (the Envelope of Unforced Noise; EUN). Typically, the EUN is derived from climate models themselves, but climate models might not accurately simulate the correct characteristics of unforced GMT variability. Here, we simulate a new, empirical, EUN that is based on instrumental and reconstructed surface temperature records. We compare the forced GMT signal produced by climate models to observations while noting the range of GMT values provided by the empirical EUN. We find that the empirical EUN is wide enough so that the interdecadal variability in the rate of global warming over the 20^th^ century does not necessarily require corresponding variability in the rate-of-increase of the forced signal. The empirical EUN also indicates that the reduced GMT warming over the past decade or so is still consistent with a middle emission scenario's forced signal, but is likely inconsistent with the steepest emission scenario's forced signal.

Change in global mean surface air temperature (GMT) can result from external radiative forcings, which impose an energy imbalance at the top of the Earth's atmosphere (e.g., changes in greenhouse gas concentrations, sulfate aerosol loading, total solar irradiance, land use albedo, etc.)^1^, as well as from unforced variability (also referred to as internal variability) that arises spontaneously from the internal dynamics of the ocean-atmosphere system^2^,^3^,^4^. This unforced variability in GMT, which is relatively chaotic and unpredictable beyond several years^5^, can result from an exchange of heat between the ocean and atmosphere^6^,^7^,^8^,^9^ and/or from an internally generated change in the earth's top-of-atmosphere energy budget^10^. In the context of societal concerns regarding global warming, it can be useful to consider the component of GMT change that is due to external radiative forcings as the ‘signal' and the component due to chaotic unforced variability as ‘noise' and thus we adopt this convention here.

Coupled Atmosphere-Ocean General Circulation Models (CGCMs) are the most common tools used to estimate how GMT responds to external radiative forcings. However, a single externally forced CGCM run will contain both a forced signal and unforced noise produced by the CGCM. The unforced noise produced by multiple runs of CGCMs with different initial conditions will be independent so the forced signal of a CGCM can be isolated by averaging over many CGCM ensemble members. Thus, in this study we use the multi-model mean across numerous CGCMs from the Coupled Model Intercomparison Projection – Phase 5 (CMIP5)^11^ (Supplementary Table S1) to represent the estimate of the forced GMT signal that we compare to observations.

The observed GMT contains both a forced signal component as well as an unforced noise component. Therefore, when the forced signal produced by CGCMs is compared to the observed GMT, it is important to consider the probability distribution of GMT values that would have been possible had the climate system been in a different unforced state. We refer to the probability distribution of GMT values that could come about due to unforced variability as the Envelope of Unforced Noise (EUN). Note that the EUN always represents a range of variability about some background GMT value that is fundamentally controlled by the external radiative forcings.

Several recent studies have compared observed GMT anomalies (and trends) with the forced signal and EUN produced by an ensemble of CGCM runs^12^,^13^,^14^,^15^. It should be noted that when different CGCMs are incorporated into the ensemble, the spread of GMT values samples uncertainty in model parameters and structure as well as uncertainty in the state of unforced variability^16^. The observed GMT anomaly in 2013 was near the lower boundary of the 5–95% EUN simulated by the CGCMs^17^ (Supplementary Fig. S1). This has been interpreted as evidence that the CGCM-simulated forced signal may be increasing too rapidly, possibly because the increase in external forcings have been overestimated^15^,^18^,^19^,^20^, and/or because the CGCMs are oversensitive to external forcings^21^,^22^. However, it has also been noted that when the CGCMs' EUN is considered, the recently observed rate of warming may still be consistent with the forced signal produced from the CGCMs^13^,^15^,^18^,^23^,^24^,^25^. Hypothetically, if the observed GMT anomaly were to fall below the CGCM-produced EUN, it would not necessarily indicate that the forced signal was increasing too rapidly. Instead, it could indicate that the CGCM-produced EUN is not large enough (i.e., that CGCMs underestimate the magnitude of unforced noise but not the magnitude of the forced rate of warming). On the other hand, if the CGCM-produced EUN is too large (i.e., if CGCMs overestimate the magnitude of unforced noise compared to reality), then recent observations may already confirm that the forced signal over the 21^st^ century is increasing too rapidly.

The unforced noise produced by CGCMs is an emergent property of the simulations and is not guaranteed to accurately represent empirical observations. Indeed, GMT variability on interannual timescales is heavily influenced by the El-Nino/Southern Oscillation (ENSO)^13^,^26^ and many CGCMs still struggle with the precise magnitude and spectral characteristics of ENSO variability^27^. Additionally, several studies have suggested that CGCMs may systematically underestimate the magnitude of interdecadal unforced variability compared to the real climate system^9^,^28^,^29^,^30^,^31^,^32^,^33^. Given that it is possible that the current generation of CGCMs may not fully capture the nature of unforced variability present in reality, it is valuable to derive an EUN from an independent source^34^,^35^.

In this study we create an empirical EUN that is based on unforced noise inferred from instrumental and reconstructed surface temperature datasets over the past millennium. We then use this EUN to revisit two topics that are relevant to the interpretation of recent climate change: 1) Is the interdecadal variability in the rate of global warming over the 20^th^ century (i.e., warming from the 1910s-1940s, a hiatus from the 1940s-1970s and resumed warming from the 1970s-2000s) large enough so that it necessitates corresponding changes in external radiative forcings? Alternatively, could this variability be the result of unforced noise superimposed on a more-or-less constantly increasing forced signal? 2) Does the reduced rate of global warming over the beginning of the 21^st^ century indicate that the CMIP5 CGCM-produced forced signal is increasing too rapidly or are hiatus periods like this more-or-less inevitable?

For the purpose of answering these questions, we create an Ensemble of Stochastic Realizations of Unforced Noise (ESRUN) which is a collection of thousands of synthetic time series produced using 2^nd^ order autoregressive, AR(2), noise models (see Methods). The high frequency (interannual to decadal) variability in these time series is based on the instrumental GMT record, which extends from the present to the late 19^th^ century. Unfortunately, the instrumental record may be insufficient to serve as the basis for low frequency (interdecadal to centennial) variability in the ESRUN because it is too short to properly characterize this variability. Additionally, the instrumental record overlaps with the era of strong anthropogenic radiative forcing. This overlap has created ambiguity as to whether observed interdecadal variability in GMT during the 20^th^ century is attributable to corresponding variations in external forcings or to unforced noise. Some studies emphasize the role of time-varying external forcings^36^,^37^,^38^,^39^,^40^ while others stress the role of unforced noise^9^,^28^,^30^,^41^,^42^,^43^,^44^,^45^,^46^,^47^,^48^. For these reasons, the ESRUN combines high frequency variability based on the instrumental record with low frequency variability based on reconstructions of surface temperature from the year 1000 to 1850.

Surface temperatures from 1000 to 1850 were influenced less by external radiative forcings than the surface temperatures during the industrial era but they nevertheless contain an externally forced component^49^,^50^. Therefore, any variability that is attributable to external forcings must be removed from the reconstructions before they can serve as a basis for the low frequency component of the ESRUN. Removal of the forced signal could theoretically be achieved by simply subtracting an estimate of the forced signal (represented by an energy balance climate model or the mean of a CGCM ensemble) from the reconstructed temperature time series^37^,^50^. However, some forced temperature changes (e.g., those due to large volcanic eruptions) can be underestimated in reconstructions^49^,^51^. In this case, subtraction of a model-calculated forced signal from a reconstruction would cause some forced variability to leak into the resulting estimate of unforced noise. In order to avoid this problem, we remove the forced component of variability from reconstructions with Multiple Linear Regression (Fig. 1a, 1b, and 1c; Supplementary Fig. S2a, S2b, and S2c; Methods) which effectively scales the forced response to the surface temperature dataset. Although many aspects of the climate system are fundamentally non-linear, several studies have indicated that the GMT response to external radiative forcings is indeed nearly linear and thus the removal of the forced signal with linear regression is justified^34^,^47^,^52^,^53^,^54^ (see also Test of Methodology in the Supplementary Material).

There is considerable uncertainty around both the reconstructed surface temperature anomalies as well as the reconstructed forcings from the year 1000 to 1850. We account for this uncertainty by sampling different combinations of estimated forcing (Fig. 1a) and surface temperature (Fig. 1b) time series in our creation of different estimates of historical unforced GMT noise (U_recon_ time series) (Fig. 1c). Additionally, many reconstructions of surface temperature over the past millennium are limited to a single hemisphere and there are few reconstructions of GMT. However, analysis of CGCMs indicates that information on GMT variability can be gleaned from the variability of a single hemisphere (Supplementary Fig. S4). In particular, these CGCMs indicate that GMT variability tends to originate relatively coherently from both hemispheres (often due to the Interdecadal Pacific Oscillation which spans both the northern and southern Pacific Ocean)^33^. Therefore, larger variability in one hemisphere tends to imply larger GMT variability (Supplementary Fig. S4). Thus, we use the information from the hemispheric reconstructions to infer GMT variability by converting hemispheric estimates of unforced noise into estimates of unforced GMT noise with scaling factors derived from CGCMs (Supplementary Table S1, Methods). Sampling uncertainty in the reconstructed surface temperatures, reconstructed forcings, and the conversion of hemispheric to global variability, yielded an ensemble of 15,120 U_recon_ time series, each representing a different possible progression of low frequency unforced GMT anomalies from the years 1000 to 1850 (a representative sample of these U_recon_ time series are shown in Fig. 1d). The ESRUN consisted of AR(2) models that simulated low frequency variability based on each of the 15,120 U_recon_ time series and combined that with AR(2) models that simulated high frequency variability based on the instrumental record (Supplementary Fig. S3, Methods). The ESRUN was then used to create a EUN characterized by the 0.5–99.5%, 2.5-97.5%, and 5-95% GMT ranges across all of the stochastic time series as a function of time (Fig. 1e).

## Results

**Figure 1:**
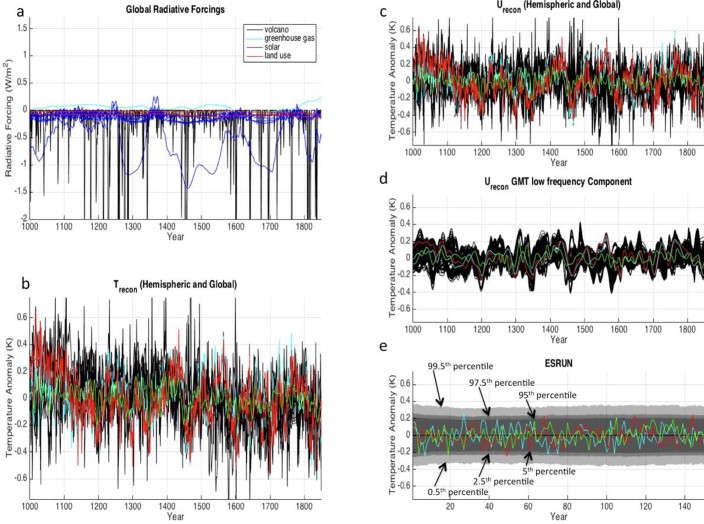
Steps involved in the creation of the low frequency component of the ESRUN. (a), External radiative forcing estimates over the period of 1000–1850 broken down into components from volcanic aerosols (2 estimates), greenhouse gas concentration (1 estimate), total solar irradiance (7 estimates) and land-use albedo (2 estimates). Note that combining these forcing reconstructions yields 2×1×7×2 = 28 possible estimates of total radiative forcing over this period. **(b),** 15 reconstructions of surface temperature (both hemispheric and global) over the period 1000–1850 with three arbitrary reconstructions colored. **(c),** Estimates of the unforced component of variability over the time period (U_recon_) which were obtained after Multiple Linear Regression was used to remove the forced variability from the reconstructions (see Methods). The same three arbitrary reconstructions are colored. Because there were 28 forcing estimates and 15 surface temperature estimates, there were 28×15 = 420 different U_recon_ estimates. **(d),** A sample of the low frequency component of the U_recon_ time series after all hemispheric estimates of unforced noise had been converted to estimates of unforced GMT noise (see Methods). The same three arbitrary reconstructions are colored. **(e),** The ESRUN, which combines AR(2)-simulated low frequency variability (based on the time series in panel d) with AR(2)-simulated high frequency variability based on the instrumental record (Figure S2, Methods). The grey shading in the ESRUN represents the width of the EUN at the three percentile intervals labeled in the panel. Three arbitrary realizations of GMT from the ESRUN are colored. Note that the timescale in panel e has changed because the ESRUN only needs to be 150 years long in order to study GMT change between 1900 and 2050 (Fig. 2 and Fig. 3).

**Figure 2:**
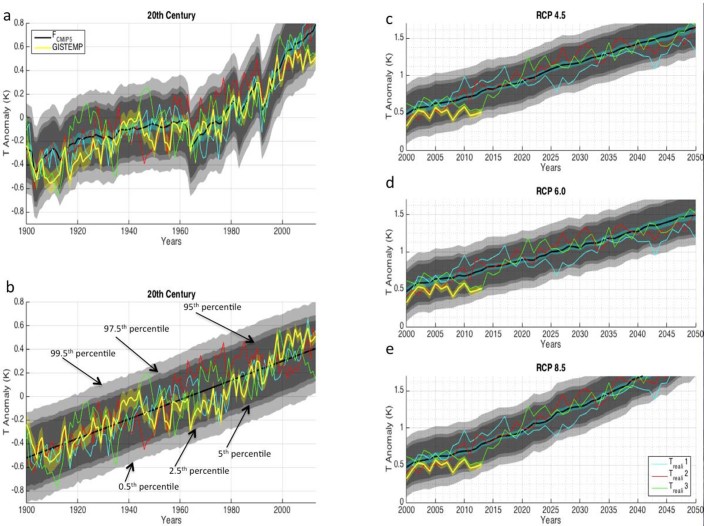
Empirical EUN superimposed on various forced signals (black lines) over the 20^th^ and 21^st^ centuries. **(a),** Forced signal originating from the multi-model mean of the CMIP5 ‘historical experiment' from 1900 to 2005 and RCP 6.0 from 2005 to 2013. **(b),** Hypothetical forced signal represented by a linear trend fit to observations during 1900 to 2013. **(c), (d) and (e),** Same as **(a)** but with forced signals corresponding to the RCP 4.5, RCP 6.0 and RCP 8.5 emissions scenarios extended to 2050. Uncertainty in the CGCM-produced forced signals is represented by ±2 standard errors calculated across all CGCM realizations (light blue shading surrounding the black line) and the EUN ranges are expanded by this uncertainty. Note that the light blue shading does not represent the uncertainty in the radiative forcings themselves but rather the uncertainty in the forced GMT signal given an assumed radiative forcing trajectory. Three arbitrary realizations of GMT from the ESRUN (superimposed on the forced signals) are shown in each panel (labeled T_reali_ 1, T_reali_ 2 and T_reali_ 3). In all plots the observed GMT^55^ (as well as its 2σ uncertainty), is shown in yellow. All anomalies are defined relative to the average from 1961–1990.

Figure 2a shows the empirical EUN (from Fig. 1e) superimposed on the forced signal produced by the CMIP5 ‘historical experiment' multi-model mean (extended to 2013 with RCP6.0). The observed GMT^55^ values fall mostly within the 2.5–97.5% superimposed EUN over the course of the 20^th^ century, indicating that observations are consistent with the ‘historical experiment' representation of the forced signal. However, the ESRUN also illustrates how different the observed GMT progression might have been over the 20^th^ century if unforced modes of variability had been in different phases. In particular, the EUN is large enough so that the early century interdecadal variability (i.e., accelerated warming from the 1910s to the 1940s and a global warming hiatus from the 1940s to the 1970s) could have come about mostly due to unforced noise. For example, the path of an arbitrary synthetic time series from the ESRUN (T_reali_ 2) shows a possible progression of cooling from the late 1920s through the mid-1940s and then sizeable warming from the mid-1940s through the 1970s and beyond.

In contrast to the decadally-varying forced signal produced in the CMIP5 ‘historical experiment' (Fig. 2a), some studies have suggested that the forced signal over the twentieth century increased in a relatively steady and monotonic manner^45^,^47^. This would have been possible if the negative forcing from anthropogenic aerosols proportionately offset the rapid increase in the positive forcing from greenhouse gasses since the mid-20^th^ century. Under this view, the observed multidecadal GMT variability would be attributed to unforced noise instead of variability in the forced signal. The empirical EUN produced in this study can be used to test if this description of the forced signal is consistent with the observed GMT progression. In Fig. 2b we display the EUN superimposed on a linear trend fit to observed GMT (ordinary least squares) during the period of 1900–2013. In this case, the observed GMT again falls mostly within this 2.5–97.5% EUN. Five out of 113 years, or ~4% fall outside the 2.5–97.5% EUN, which is about the proportion expected by random chance. This suggests that the stepwise acceleration of GMT warming over the 20^th^ century (i.e., acceleration from ~1910–1940, deceleration until ~1975, acceleration until ~2000) does not necessarily require corresponding deviations in the rate of increase of the forced signal and could have come about due to unforced noise. This is consistent with the notion that a non-trivial portion of the GMT warming between the mid-1970s and mid-2000s may have been due to unforced noise^9^,^41^,^45^,^47^.

Most radiative forcing scenarios project a monotonic increase in the global warming signal throughout the 21^st^ century. However, the unforced noise revealed in this analysis would leave open the possibility of significant deviations from this long-term GMT ascent. Figures 2c, 2d, and 2e show the empirical EUN superimposed on the CMIP5 multi-model mean incorporating Representative Concentration Pathways (RCPs) 4.5, 6.0 and 8.5^56^ respectively. It should be noted that RCP 4.5 actually increases at a faster rate than RCP 6.0 over the first half of the 21^st^ century. It is apparent that despite the monotonically increasing forced signal, the ESRUN illustrates that large deviations from the forced signal can persist for more than a decade. For example, T_reali_ 1 shows a cooling trend from 2016 to 2028 and T_reali_ 3 shows a warming rate from 2013 to 2024 that is more than double that attributable to the forced signal. These deviations illustrate that GMT trends calculated over a decade or two may say very little about the underlying forced signal and thus should be interpreted with caution.

Despite the notable magnitude of unforced variability apparent in the ESRUN, the year 2013 in the observational record lies near the lower boundary of the 2.5–97.5% EUN for all three RCP scenarios. Note that GISTEMP was used to represent observations because it has the most extensive spatial coverage of the three main surface temperature datasets and thus is least affected by the shortage of observations in the Arctic^55^. The exact location of the EUN border relative to observations, however, is somewhat dependent on the arbitrarily chosen time period over which the anomalies are defined (in this case 1961–1990). Therefore, a more meaningful way to assess the recently observed GMT progression relative to the CGCM-produced forced signals would be to compare recent GMT trends to the probability distribution of trends that occurred within the ESRUN superimposed on the forced signals. All three forced signals (corresponding to the three main RCP forcing scenarios) are included in this analysis as it is possible that the recent forced signal could have followed any of their trajectories. This is primarily because of large uncertainty in the magnitude of negative anthropogenic aerosol forcing^57^.

**Figure 3:**
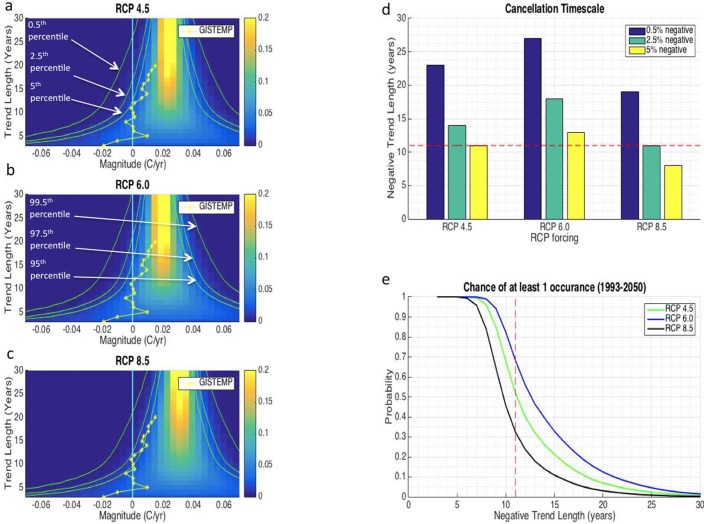
Probabilities of observing trends of indicated lengths and magnitudes associated with the CGCM-produced forced signals incorporating the RCP 4.5, RCP 6.0 and RCP 8.5 emission scenarios. **(a), (b)** and **(c)** show the probability of observing a linear trend of a given length and within the given magnitude bin from 1993 to 2050 as indicated by superimposing the ESRUN on the RCP 4.5, RCP 6.0 and RCP 8.5 forced signals respectively. The yellow line represents the observed trends in the GISTEMP dataset ending in 2013 and progressing backwards in time for the length of the trend (e.g., the yellow star associated with the 15-year trend length represents the linear trend from 1998 to 2013). This comparison helps indicate whether or not the observed rate of warming from 1993–2013 is consistent with the mean rate of warming from 1993–2050 expected from the 3 CGCM-produced forced signals. **(d),** Cancellation Timescale (see text for definition) at 0.5%, 2.5% and 5% for RCP 4.5, RCP 6.0 and RCP 8.5 **(e),** probability of observing at least 1 negative linear trend of the given length over the period 1993–2050 (see Methods). The red dashed line in both **(d)** and **(e)** represents the negative 11-year trend that was observed in GISTEMP from 2002 to 2013. All trends were calculated with an ordinary least squares procedure.

Figures 3a, 3b, and 3c show the linear trend distributions of the ESRUN superimposed on the three RCP forced signals, as a function of trend length. These distributions account for all overlapping trends of a given length between the years 1993–2050. 1993 was chosen as the start year in order to extend the analysis as far back as possible while still avoiding any major volcanic eruptions and 2050 was chosen as the end year to avoid the post-2050 reduction in the rate of increase of the RCP 4.5 forced signal^56^. These trend distributions are compared to the recently observed trends calculated on the GISTEMP dataset from 1993–2013, 1994–2013, … , 2010–2013. The goal of this comparison is to see if observed trends over the 1993–2013 period are consistent with the mean rate-of-increase of the CGCM-produced forced signals between 1993–2050.

No trend calculated on the observational record since 1993 falls outside the 2.5–97.5% trend EUNs for any of the three forced signals (Fig. 3a, 3b, and 3c). If we consider the trend EUNs to be null distributions and the observed trends to be test statistics, then this result indicates that we would not be able to reject any of the forced signals at the 95% significance level. However, trends from 8–20 years in length are all near or outside the 5–95% trend EUN for the RCP 8.5 forced signal, indicating that over the most recent decade or two, it is unlikely that we have experienced an increase in the forced signal as steep as the mean rate-of-increase of the forced signal for RCP 8.5 between 1993–2050.

Over the years 2002–2013, a linear trend fit to GISTEMP reveals a slightly negative slope (Figure 3a, 3b, 3c). Because special attention is often given to cooling episodes embedded in the long-term global warming signal, it is valuable to quantify the likelihood of such events occurring. To serve this purpose, we define the Cancellation Timescale, which is the longest period of time where there is at least a small chance (5%, 2.5% or 0.5%) that the positive trending forced signal could be completely ‘canceled out' by a negative unforced fluctuation, resulting in a flat trend line. The Cancellation Timescale in Figs. 3a, 3b and 3c is the highest point on the vertical axis (i.e., longest timescale) where the 5^th^, 2.5^th^ and 0.5^th^ percentile lines cross the vertical zero-magnitude line. The Cancellation Timescales for all three RCPs are shown in Fig. 3d. The 11-year negative trend in observations from 2002–2013 would need to extend until 2015 before it would be considered outside the 5^th^ percentile of trends associated with the RCP 6.0 forced signal, indicating that it would be premature to conclude that the forced signal is increasing at a slower rate than the RCP 6.0 forced signal (Fig. 2d). However, a negative trend of 11 years matches the 2.5% Cancellation Timescale and exceeds the 5% Cancellation Timescale (8 years) associated with the RCP 8.5 forced signal, indicating that we may be able to reject this rate of forced warming at the 97.5% confidence level. For the RCP 8.5, 6.0, and 4.5 forced signals, the 0.5% Cancelation Timescales are 19 years, 27 years and 23 years respectively. This would indicate that the global warming hiatus would need to continue for 8–16 years beyond 2013 before it could be said with over 99% confidence that the true forced signal is not increasing as quickly as these CGCM-produced forced signals.

The null distributions in Figs. 3a, 3b, and 3c give us information about how likely a trend of a given length and magnitude would be if it were randomly selected from the ESRUN superimposed on the forced signals. However, the most recent negative trend was not randomly selected. Instead, the lack of observed warming from 2002–2013 helped motivate this study in the first place. This could introduce a selection bias as we may have waited for an extreme trend to occur before we calculated how likely the extreme trend was in the first place. Because extremes are inevitable in the long run, this would increase the chance of committing a type – 1 error (i.e., the null hypothesis is mistakenly rejected). Thus, in addition to the Cancellation Timescale, a complimentary measure of the likelihood of the current warming hiatus is the chance of observing at least 1 negative linear trend over the entire period from 1993–2050 (Fig. 3e). We find that a negative linear trend of 11 years is not extremely unlikely in any of the forced signal trajectories over this time period. In fact, for the RCP 6.0 forced signal, there is a ~70% chance of seeing at least one negative linear trend of 11 years or longer between 1993–2050 (see Methods). On the other hand, there is only a ~30% chance of seeing at least one negative linear trend of 11 years or longer for the RCP 8.5 forced signal over the same period. This further supports the notion that the lack of warming observed over the past decade or so is not inconsistent with the RCP 6.0 forced signal but that it is unlikely to have occurred under the RCP 8.5 forced signal. This may be because the external forcings have not increased as fast over the recent decade as was assumed in the RCP 8.5 emission scenario^15^,^18^,^19^,^20^.

### Summary

In this work we created a very large ensemble of stochastic realizations of unforced GMT noise that were based empirically on the instrumental record and reconstructions of surface temperature over the past millennium. We used this ensemble to create an empirical estimate of the EUN and used it in the comparison between observations and the forced signal produced by CGCMs over the 20^th^ and 21^st^ centuries. We find that the interdecadal variability in the rate of global warming over the 20^th^ century (i.e., acceleration from ~1910–1940, deceleration until ~1975, acceleration until ~2000) is within the 2.5–97.5% EUN, even if the forced signal is represented as a linear trend, indicating that this observed interdecadal variability in the rate of warming does not necessarily require interdecadal variability in the rate-of-increase of the forced signal. We also find that recently observed GMT values, as well as trends, are near the lower bounds of the EUN for a forced signal corresponding to the RCP 8.5 emissions scenario but that observations are not inconsistent with a forced signal corresponding to the RCP 6.0 emissions scenario.

## Methods

### The Ensemble of Stochastic Realizations of Unforced Noise (ESRUN)

The ESRUN is an ensemble of 15,120 unique time series that represent hypothetical realizations of unforced GMT noise over an arbitrary 150-year period. The ESRUN is thus analogous to an ensemble of CGCM runs that differ in their initial conditions and other model parameters that might affect the characteristics of unforced noise. Unlike a CGCM ensemble, however, the ESRUN simulates variability that is empirically based on the instrumental record as well as reconstructed surface temperatures over the past millennium. The time series that make up the ESRUN are stochastic since they are based on 2^nd^ order autoregressive, AR(2), noise models that incorporate random number generation. Therefore, the time series in the ESRUN are intended to simulate the characteristics (i.e., magnitude and spectral features) of unforced modes of variability apparent in the instrumental and reconstructed surface temperature time series but they are not expected to be in phase with any historical variability.

The AR(2) models that make up the time series in the ESRUN are fit to Intrinsic Mode Functions (IMFs) that result from Empirical Mode Decomposition^58^ being applied to the instrumental and reconstructed surface temperature datasets. Time series were split into their component IMFs so that the AR(2) models could simultaneously simulate the high frequency variability apparent in the instrumental record and the low frequency variability apparent in reconstructed time series. High frequency variability was defined as any IMF with a mean wavelength of 15 years or less (two or more zero crossings per 15 year period on average) and low frequency variability was defined as any IMF with a mean wavelength larger than 15 years (less than 2 zero crossings per 15 year period on average). We chose 15 years as the division between high and low frequency variability because this serves as a natural division between ENSO variability (which has a characteristic timescale of ~3–7 years) and slower evolving modes of variability such as the Atlantic Multidecadal Oscillation and Interdecadal Pacific Oscillation which have timescales of multiple decades^59^. See the ‘Division Between High and Low Frequency Variability' section of the Supplementary Material for further discussion of the 15-year threshold.

**Figure 4:**
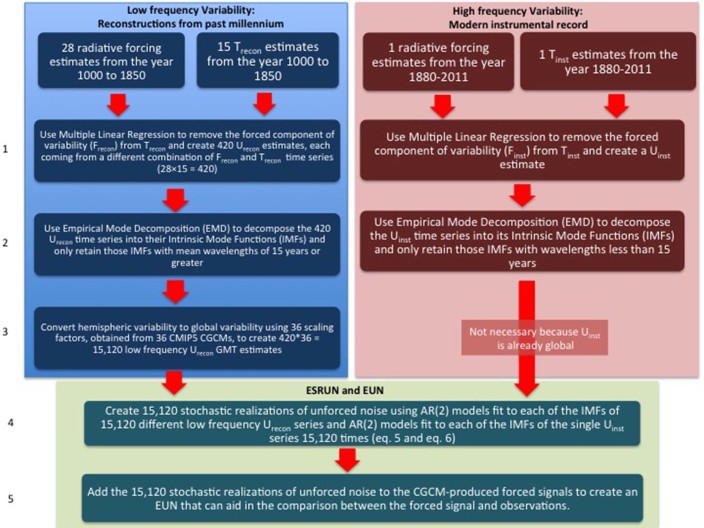
Flow chart illustrating the steps (labeled on the left) associated with the creation of the empirically-based Ensemble of Stochastic Realizations of Unforced Noise (ESRUN). Steps concerning the reconstructed surface temperatures and forcings are in blue while steps concerning the surface temperatures and forcings over the instrumental era are in red. Steps dealing with combing of information from reconstructions and instrumental data are in green.

We base high frequency variability in the ESRUN on the instrumental record because the instrumental record represents the highest quality data available and it is of sufficient length to statistically characterize variability at timescales of 15 years and less. However, the instrumental record may be of insufficient length to statistically characterize some modes of low frequency variability. Additionally, there is ambiguity as to whether some low frequency variability apparent in the instrumental record is attributable to unforced modes or to time-varying external radiative forcings. For these reasons, the low frequency variability in the ESRUN is based on reconstructions of surface temperature from the year 1000 to 1850. The specific steps involved in creating the ESRUN are described below and illustrated in Fig. 4.

#### 1) Isolating Unforced Variability via Multiple Linear Regression

We make the simplifying assumption that the instrumental (T_inst_) and reconstructed (T_recon_) surface temperature time series are linear combinations of forced (F_inst_, F_recon_) and unforced (U_inst_, U_recon_) variability,


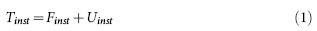

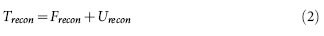


The forced component of variability is represented as the linear combination of various external forcings, 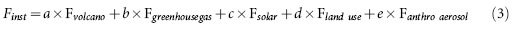

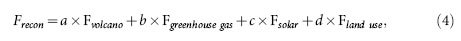


where the coefficients a, b, c, d, and e are obtained by fitting the forcing time series (Fig. 1a and Supplementary Fig. S2a) to T_inst_ and T_recon_ using Multiple Linear Regression (MLR) via ordinary least squares. U_inst_ and U_recon_ are then obtained by subtracting F_inst_ and F_recon_ from T_inst_ and T_recon_, respectively (Fig. 1b and Supplementary Fig. S2b). Therefore, the variability that is *not* explained by the forcings (the residual in the MLR procedure) is considered to be the unforced variability (Fig. 1c and Supplementary Fig. S2c). Removing the forcing with MLR rather than using physical models allows for the forcing to be scaled to the observed/reconstructed temperature and thus prevents forced variability from leaking into the estimate of the unforced variability. This methodology seems justified since several studies have indicated that the large-scale surface temperature response to external radiative forcings is nearly linear and thus the forced signal should be able to be removed from the instrumental and reconstructed datasets with MLR^34^,^47^,^52^,^53^,^54^. See the Test of Methodology section in the Supplementary Material for a demonstration of MLR's ability to remove the forced signal from the record.

In order to sample the uncertainty in both F_recon_ and T_recon_, 28 different global external forcing time series combinations (Fig 1a) from Schmidt, et al.^60^ and 15 different T_recon_ time series from Wahl, et al.^61^ (Fig. 1b, Supplementary Material) were used to create 15×28 = 420 U_recon_ estimates via equations (2) and (4). The 28 F_recon_ time series represented all possible F_recon_ time series from Schmidt, et al.^60^ and the 15 T_recon_ time series represented all T_recon_ time series from Wahl, et al.^61^ with at least hemispheric spatial scale and annual temporal resolution. We did not sample uncertainty over the instrumental time period (F_inst_ and T_inst_) because GMT uncertainty is much smaller (shown in yellow shading in Fig. 2) over the instrumental time period than it is in the reconstructions from 1000–1850 and the high frequency component of variability (red box in Supplementary Fig. S2) is not sensitive to uncertainty in forcings over the instrumental time period. This is because most of the uncertainty in the forcing over the instrumental time period is associated with the slowly-changing anthropogenic aerosol forcing which does not project onto the variability at timescales of 15 years or less.

#### 2) Decomposing Variability by Timescale

U_recon_ and U_inst_ were both split into their Intrinsic Mode Functions (IMFs) via Empirical Mode Decomposition (EMD)^45^,^58^,^62^. It was necessary to split the time series into their IMFs so that the ESRUN would be able to simultaneously simulate low frequency variability based on the U_recon_ time series and high frequency variability based on the U_inst_ time series (Supplementary Fig. S3). EMD also had the additional benefit of being able to conserve multiple modes of variability at a single time. This is advantageous because the real climate system will have several internal modes effecting GMT at any given time (e.g., the Atlantic Multidecadal Oscillation and the Interdecadal Pacific Oscillation). See the Test of Methodology section in the Supplementary Material for a demonstration of EMD's ability to conserve physical modes of variability in a CGCM's control run.

#### 3) Inferring GMT Variability from Hemispheric Variability

Supplementary Fig. S4 indicates that information on GMT variability can be inferred from the variability of a single hemisphere, albeit with an added element of uncertainty. Since this study is concerned with GMT variability, any low frequency U_recon_ IMFs that represented hemispheric rather than global variability were scaled to convert them into representations of GMT variability. Thirty-six scaling factors were obtained via an investigation of the ratio of hemispheric to GMT variability (measured with the standard deviation) in 36 CMIP5 CGCM unforced control runs (Supplementary Table S1 and Supplementary Fig. S4). The spread of these 36 scaling factors represents uncertainty in the conversion from hemispheric to global variability. In order to sample this uncertainty, each U_recon_ time series was converted to global variability once for each of the 36 scaling factors, making 420×36 = 15,120 total U_recon_ time series of low frequency GMT (Fig. 1d). If a U_recon_ time series was already global in coverage, then 36 scaling factors of 1 were used so that the time series would maintain the same proportion in the resulting ensemble of 15,120 that it had in the original ensemble of 420 time series. See the Test of Methodology section in the Supplementary Material for a demonstration of the effectiveness of converting hemisphere surface temperature to GMT.

#### 4) AR(2) Noise Modeling of Unforced GMT Variability

AR(2) noise models were fit (using the Yule-Walker method) to each low frequency IMF of each of the 15,120 U_recon_ time series,


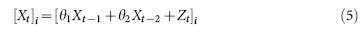


where X_t_ is the IMF value for any given year; X_t-1_, X_t-2_ are the IMF values for the previous two years; θ_1_ and θ_2_ are autoregressive coefficients, Z_t_ is a normal random variable and the subscript i indicates the particular IMF that the AR(2) model was fit to (Supplementary Fig. S3).

Similarly, AR(2) noise models were fit to each high frequency IMF of the single U_inst_ time series (Supplementary Fig. S3). 15,120 high frequency stochastic AR(2) time series were created but since they were all fit to the same three U_inst_ IMFs they were not sampling uncertainty in forcings and temperatures and only differed in their Z_t_ values.

The low frequency AR(2) IMF simulations based on the U_recon_ series and the high frequency AR(2) IMF simulations based on the U_inst_ series were then summed to produce stochastic realizations of unforced GMT variability,


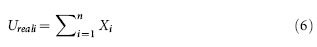


where n is the number of high frequency instrumental IMFs (3, see Supplementary Fig. S2 and S3) plus the number of low frequency reconstruction IMFs that had at least 1 full oscillations over the domain length (1000 – 1850). AR(2) models did not simulate IMFs without at least 1 full oscillation because this would represent a nonstationary process at the time scale of examination (Supplementary Fig. S3). This means that the ESRUN was unable to represent variability that had wavelengths approaching the length of the reconstructions. See the Test of Methodology section for a demonstration of the ability of AR(2) simulated IMFs to represent unforced GMT variability.

AR(2) models were chosen because they are of relatively low order but still appeared capable of simulating the primary characteristics of IMF variability at the variety of timescales (Supplementary Fig. S3). Additionally, assigning a constant autoregressive model order kept the methodology simpler than it otherwise would be. Nevertheless, we also show results where the autoregressive model order was assigned separately for each IMF using Bayesian Information Criterion^63^ (Supplementary Figs. S9 and S10) as well as the results when using AR(1) and AR(7) models (Supplementary Figs. S11, S12, S13 and S14).

#### 5) Unforced Noise Superimposed on Forced Signal Over the 20^th^ and 21^st^ Centuries

The 15,120 unforced GMT noise time series resulting from Equation (6) make up the ESRUN. These estimates of unforced GMT variability were added to estimates of the CGCM-produced forced signal over the 20th and 21st centuries in order to provide context for the comparison between the forced signal and observations over that time period (Fig. 2 and Fig. 3). Figure 2's gray boundaries mark the 0.5–99.5%, 2.5–97.5%, and 5–95% boundaries of the ESRUN which we refer to as the EUN superimposed on the forced signal. We refer to individual realizations of GMT that combine forced signal and unforced noise as T_reali_ time series (Fig. 2).

### Calculation of at Least 1 Negative Linear Trend from 1993–2050

To calculate the chance of at least a single negative linear trend (of a given length) occurring between 1993–2050 (Fig. 3e), we simply count the number of T_reali_ ensemble members (in the ESRUN superimposed on the forced signal) that contained at least 1 negative linear trend of the given length and divided that count by the total number of ensemble members, 15,120.

## Author Contributions

P.T.B conceived of the study, performed the initial analysis and wrote a first draft of the manuscript. W. L., E. C. C. and S. A. M contributed to interpreting the results, discussion of the methods and refinement of the manuscript.

## Supplementary Material

Supplementary Information Supplementary Information
