# Supplementary material for: Comparing the model-simulated global warming signal to observations using empirical estimates of unforced noise
Source: Sci Rep. 2015 Apr 21;5:9957. doi: 10.1038/srep09957 (PMC4404682; doi:10.1038/srep09957)
Supplement: Supplementary Information [file srep09957-s1.pdf]

Supplementary Material

Comparing the model-simulated global warming signal to observations using empirical estimates of unforced noise

Patrick T. Brown[1*], Wenhong Li[1], Eugene C. Cordero[2], Steven A. Mauget[3],

[1] *Earth and Ocean Sciences, Nicholas School of the Environment,*

Duke University, Durham, NC 27708

[2] *Department of Meteorology and Climate Science*

San José State University, San José, CA 95192

[3] *Agricultural Research Service,*

United States Department of Agriculture, Lubbock, TX 79415-0000

Revised to: *Scientific Reports*

March 2015

[*] *Correspondence author:* Patrick T. Brown, Earth and Ocean Sciences, Nicholas School of the Environment, Duke University, 5120 Environment Hall, Durham, NC, 27708. E-mail: Patrick.Brown@duke.edu

Table S1 | CMIP5 CGCMs used in this study. 'X' marks indicate that a particular CGCM run was incorporated into the ensemble mean (representing the forced signal) in the given experiment. Also shown are the hemispheric-to-global variability scaling factors that were applied to the low frequency component of unforced hemispheric surface temperature reconstructions (Fig 1c) to convert them to representations of GMT variability (Fig. 1d). One preindustrial control run was used for each CGCM to obtain the hemisphere to global scaling factor.

Model	Historical	RCP 4.5	RCP 6.0	RCP 8.5	Preindustrial Control NH-to-Global Scaling Factor	Preindustrial Control SH-to-Global Scaling Factor
ACCESS1-0		X		X	0.60	2.26
ACCESS1-3		X		X	0.73	2.15
bcc-csm1-1	X	X	X	X	1.12	0.67
bcc-csm1-1-m	X	X	X		2.00	0.60
BNU-ESM		X		X		
CanESM2		X		X	0.62	0.73
CCSM4	X	X	X	X	0.66	0.75
CESM1-BGC		X		X	0.99	0.78
CESM1-CAM5	X	X	X	X	0.78	0.86
CESM1-FASTCHEM					0.58	0.45
CESM1-WACCM					0.74	0.99
CMCC-CESM					0.86	1.06
CMCC-CM		X		X		
CMCC-CMS		X		X	0.71	0.57
CNRM-CM5		X		X	0.64	0.56
CSIRO-Mk3-6-0	X	X	X	X	0.62	0.97
EC-EARTH		X		X		
FGOALS-g2		X		X	0.77	0.54
FGOALS-s2						
FIO-ESM	X	X	X	X		
GFDL-CM3	X	X	X	X	0.90	0.65
GFDL-ESM2G	X	X	X	X	0.66	0.88
GFDL-ESM2M	X	X	X	X	0.70	1.27
GISS-E2-H	X	X	X	X	0.87	0.53
GISS-E2-H_p3					0.86	0.84
GISS-E2-H-CC		X				
GISS-E2-R	X	X	X	X	0.91	0.86
GISS-E2-R_p2					0.63	1.44
GISS-E2-R-CC		X				
HadGEM2-AO	X	X	X	X		
HadGEM2-CC		X		X	0.85	0.88
HadGEM2-ES	X	X	X	X		
inmcm4		X		X	0.56	1.10
IPSL-CM5A-LR	X	X	X	X	0.73	1.06
IPSL-CM5A-MR	X	X	X	X	0.64	0.88
IPSL-CM5B-LR		X		X	0.66	1.13
MIROC-ESM	X	X	X	X	0.89	1.15
MIROC-ESM-CHEM	X	X	X	X	0.78	1.63
MIROC5	X	X	X	X	0.64	1.05
MPI-ESM-LR		X		X	0.78	1.03
MPI-ESM-MR		X		X	0.66	0.93
MPI-ESM-P					0.72	1.12
MRI-CGCM3	X	X	X	X	0.71	0.82
NorESM1-M	X	X	X	X	0.78	0.98
NorESM1-ME	X	X	X	X	0.73	1.30

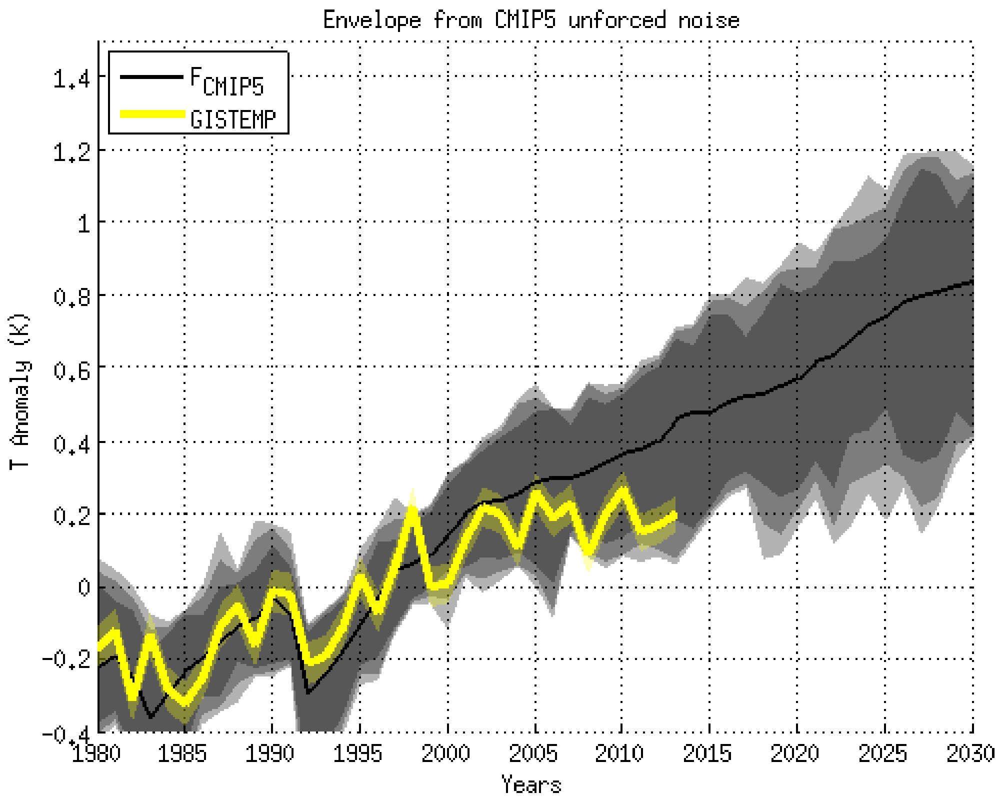

Figure S1 | CGCM produced forced global warming signal (black line) and the CGCM produced 5-95%, 2.5-97.5% and 0.5-99.5% EUNs (grey shading) of GMT anomaly values from the CMIP5 multi-model ensemble. The forced signal follows the 'historical' experiment through 2005 and the RCP 6.0 emissions scenario from 2005-2030. The GISTEMP observations (as well as their 2σ uncertainty) are shown in yellow.

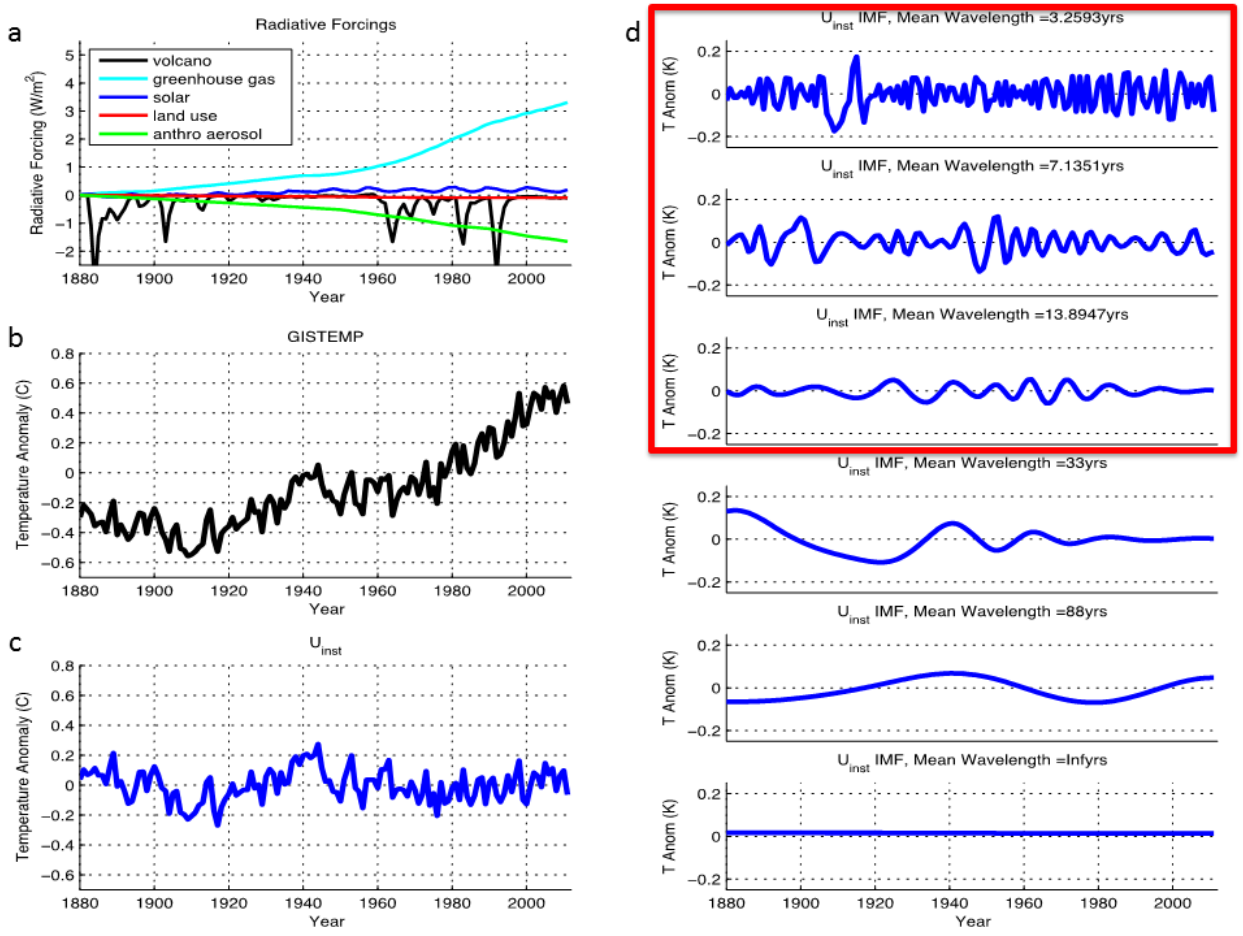

Figure S2 | Removal of the forced variability from the instrumental record from 1880-2011 and the decomposition of the unforced variability into its Intrinsic Mode Functions (IMFs). (a), External radiative forcing estimates from 1880-2011, updated from Hansen, et al. [1]. **(b),** Instrumental (GISTEMP) global mean surface air temperature from 1880-2011. **(c),** Unforced component of variability over the time period (U_{inst}) which was obtained after Multiple Linear Regression was used to remove the forced variability from the instrumental record [Equation 3 and 1]. **(d),** Intrinsic Mode Functions (IMFs) that result from Empirical Mode Decomposition[2] applied to the U_{inst} time series. Only those IMFs with a mean wavelength of 15 years or less are used in the creation of the ESRUN (red box) whereas the ESRUN bases its lower frequency variability off of reconstructions of surface temperature from the years 1000-1850 (Fig. 1 and S3).

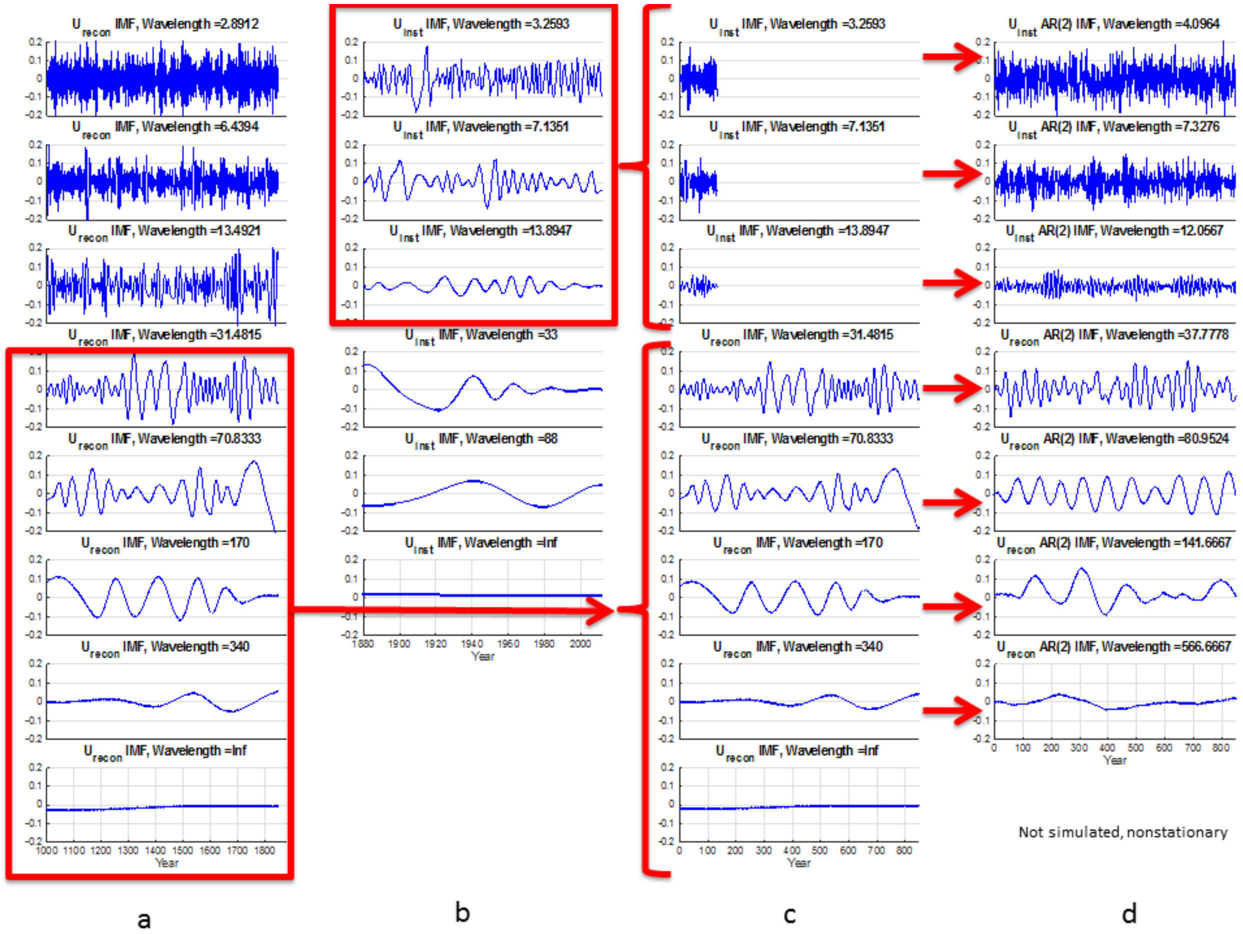

Figure S3 | Combining low frequency IMFs from reconstructions with high frequency IMFs from the instrumental record to create a stochastic realization of unforced GMT noise. (a), Intrinsic Mode Functions (IMFs) that result from Empirical Mode Decomposition[2] applied to a particular U_{recon} time series **(b),** IMFs that result from Empirical Mode Decomposition applied to the U_{inst} time series (also shown in Supplementary Fig. S2d). **(c),** High frequency IMFs from the instrumental record lined up with low frequency IMFs from the reconstructed record. At this point the low frequency variability has been converted to a representation of GMT (step 3, Methods). **(d),** AR(2) simulated IMFs that attempt to emulate the approximate magnitude and frequency, but not necessarily the phase, of the corresponding IMFs in column c.

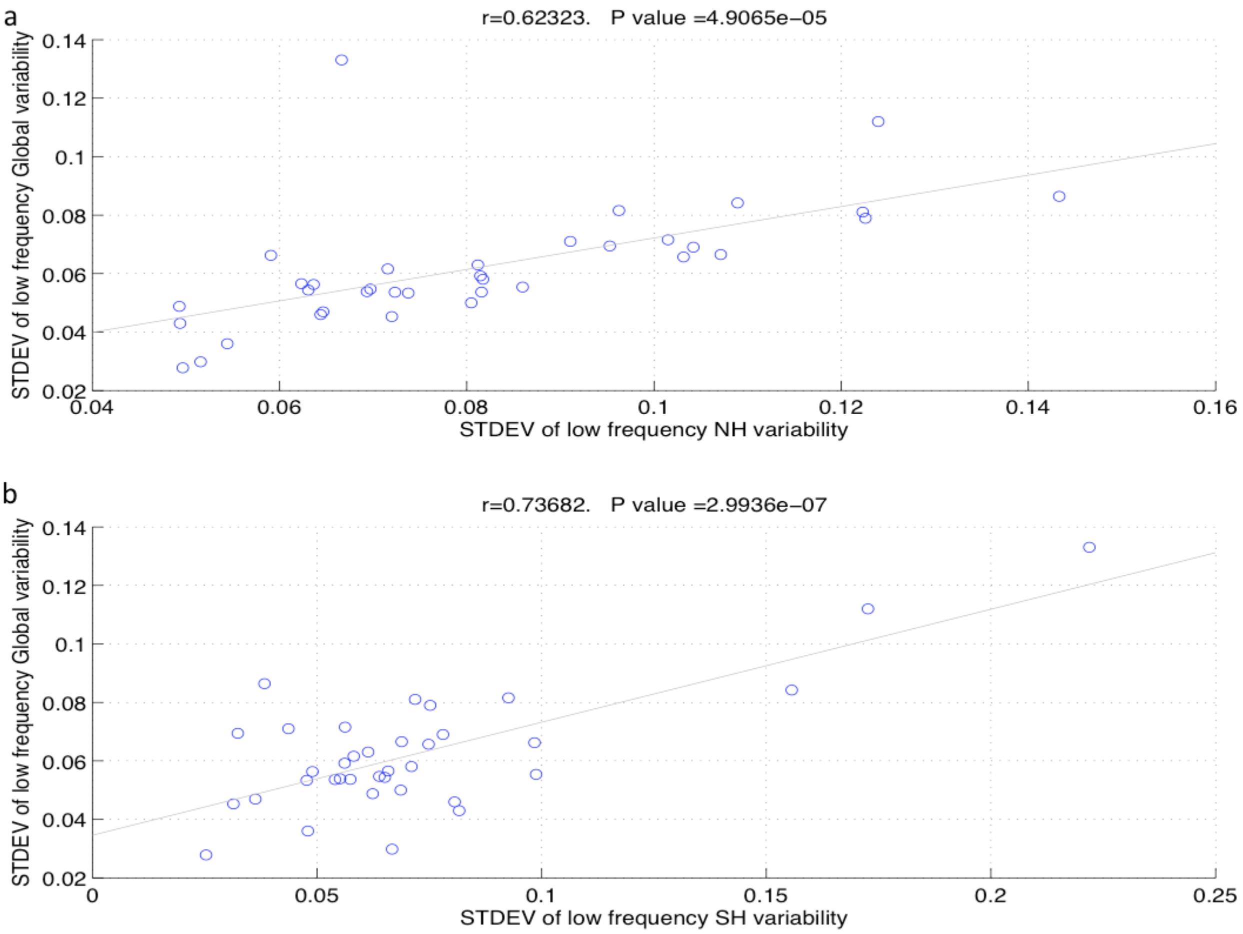

Figure S4 | Hemispheric and global variability (standard deviation) for the CMIP5 unforced preindustrial control runs (a), Relationship between the magnitude of low frequency variability of Northern Hemispheric mean surface temperature and the magnitude of GMT variability. **(b),** Same as **(a)** but comparing Southern Hemispheric variability to GMT variability. In both cases there is a statistically significant correlation (P values shown in the panels) indicating that information on unforced GMT variability can be gleaned from hemisphere mean surface temperature variability albeit with substantial uncertainty. The ratio of the y-value to the x-value, for each model, is the scaling factor listed in Table S1.

Test of methodology

Because the methodology for constructing the ESRUN has not been implemented previously, it was necessary to subject some of the fundamental concepts to basic tests in order to determine if the method was sound. For this task, a single CGCM was utilized (CSIRO-Mk3-6-0) because it had a large number of ensemble members (10) for the 'historical' experiment. We did not use the 'last millennium' experiment because no CGCMs had a large number of ensemble members for this experiment. For the following three demonstrations of concept, we treat the output of this CGCM as the "true" climate system.

1. **Conserving physical modes of unforced variability.** The ability of EMD (and subsequent AR(2) modeling of IMFs) to conserve physical modes of unforced variability on a variety of time scales was tested. This was done by simply decomposing CSIRO-MK3-6-0's unforced preindustrial control run with EMD, simulating the IMFs with AR(2) models, and summing the AR(2) simulations to create a stochastic realization of unforced GMT variability. This process was performed 100 times and the resulting synthetic time series were compared to the original unforced control run that they were attempting to emulate (considered the "true" unforced GMT variability in this experiment). The synthetic unforced GMT time series produced in this manner had a similar standard deviation (Fig. S5a), mean power spectra (Fig. S5b), and mean autocorrelation function (Fig. S5c) compared to the true unforced variability produced by the control run. This result indicates that the physical modes of variability in a CGCM's control run were conserved by the described methodology.
2. **Ability of Multiple Linear Regression to remove the forced signal.** It has been suggested that external forcings may excite and/or modulate modes of unforced variability [3-5]. This might mean that GMT could have a nonlinear response to external radiative forcings. Therefore, it was necessary to test whether it was reasonable to use Multiple Linear Regression to remove the forced signal from temperature datasets. This was done by utilizing 10 forced runs of CSIRO-MK3-6-0 over the period from 1880-2011 (historically forced from 1850-2005 and forced with RCP 6 from 2006 to 2011). In this experiment, the observed temperature was represented by GMT realizations associated with individual forced ensemble members of CSIRO-MK3-6-0 and the external radiative forcings were the same as those shown in Fig. S2. Application of Multiple Linear Regression to each of the ensemble members then produced 10 unforced GMT time series that were used to produce 100 synthetic unforced GMT realizations (10 for each) according to the ESRUN methodology. This ensemble of 100 synthetic unforced GMT realizations was compared to the "true" unforced GMT variability of the CGCM's unforced control run. Note that the synthetic unforced GMT realizations produced in this experiment were blind to the "true" unforced variability of the CGCMs control run that they are attempting to emulate. These 100 synthetic unforced GMT time series had a similar standard deviation (Fig. S5d) mean power spectra (Fig. S5e), and mean autocorrelation function (Fig. S5f) compared to the "true" unforced variability produced by the control run. This similarity indicates that Multiple Linear Regression is able to remove the forced component of variability from the record while leaving the unforced variability behind.
3. **Conversion of unforced hemispheric temperature variation to GMT variation.** The conversion from unforced hemispheric mean surface temperature variability to unforced GMT variability was also tested. This test was conducted in a similar manner to experiment 2. In this experiment, however, the observed GMT was represented by Northern Hemisphere mean surface temperature realizations associated with individual forced ensemble members of CSIRO-MK3-6-0. Application of Multiple Linear Regression then produced 10 unforced time series that were again used to produce 100 synthetic unforced realizations (10 for each) using the ESRUN methodology. In this case, however, the IMFs were converted from hemispheric variability to GMT variability with a conversion factor of

0.77 (the mean Northern Hemisphere-to-GMT conversion factor in the CMIP5 ensemble (Table S1)). These 100 synthetic unforced GMT realizations were then compared to the “true” unforced GMT variability of the unforced control run. Note that, as in experiment 2, the synthetic unforced GMT realizations produced in this experiment were blind to the “true” unforced variability of the CGCMs unforced control run that they are attempting to emulate. The 100 synthetic unforced GMT time series produced in this manner had a similar standard deviation (Fig. S5g), mean power spectra (Fig. S5h), and mean autocorrelation function (Fig. S5i) compared to the “true” unforced GMT variability produced by the control run. This similarity indicates that the conversion from Hemispheric to GMT unforced variability may be achieved through a simple scaling factor.

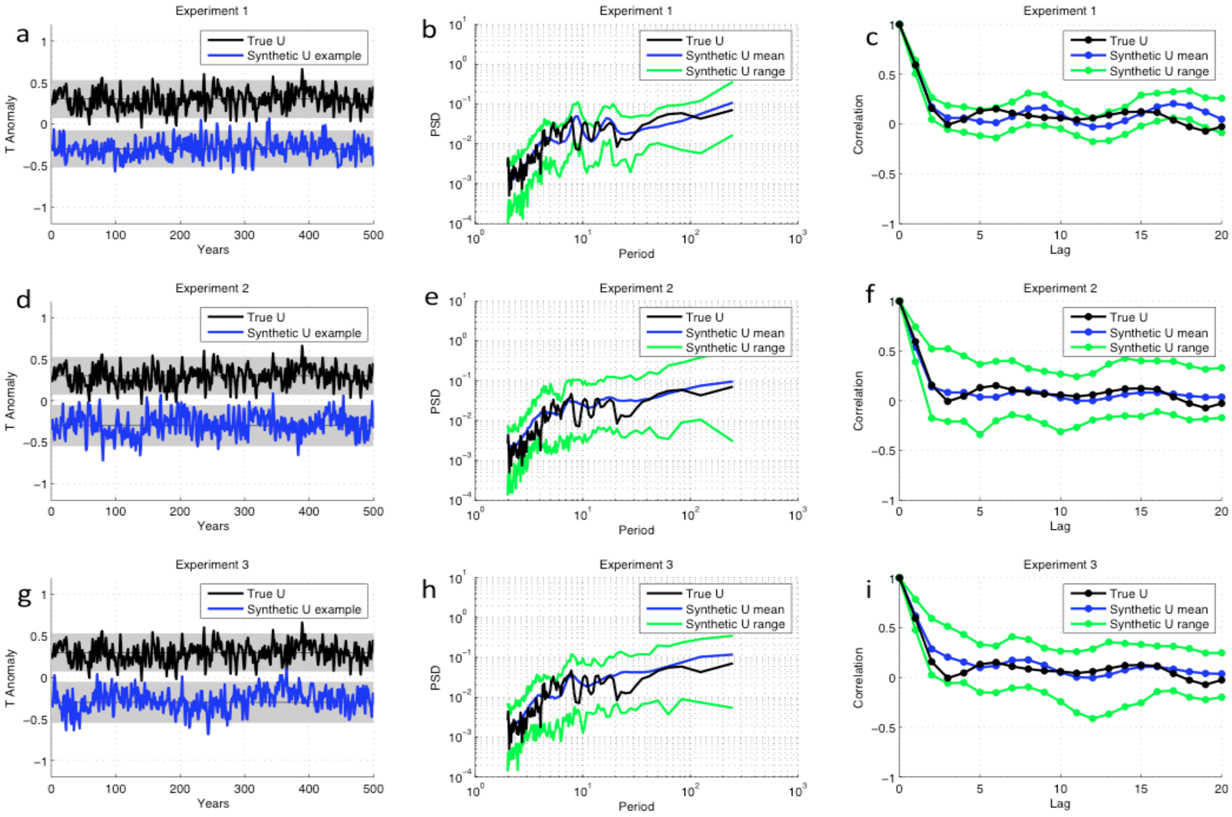

Figure S5 | Time series, multi-taper power spectra and autocorrelation function comparisons associated with methodological tests described in the text. Top row, Comparison of CSIRO-MK3-6-0 unforced control run (True U) and the unforced GMT variability produced from EMD and IMF AR(2) simulation of the same CSIRO-MK3-6-0 unforced control run (Synthetic U). **Middle**, Comparison of CSIRO-MK3-6-0 control run (True U) and synthetic unforced GMT produced from the methodology applied to 10 GMT simulations associated with forced runs of CSIRO-MK3-6-0 over the period 1880–2011. **Bottom**, Comparison of CSIRO-MK3-6-0 control run (True U) and synthetic unforced GMT produced from the methodology applied to 10 Northern Hemispheric simulations associated with forced runs of CSIRO-MK3-6-0 over the period 1880–2011. In each time series comparison (left column), a single synthetic unforced GMT realization is shown that had a standard deviation representative of the average. The time series have been shifted on the vertical axis so that they may be visually compared.

The Division between High and Low Frequency Variability

Figure S6 compares the power spectral density of unforced GMT estimated from the instrumental record and unforced GMT estimated from the reconstructions. Reconstructions where not used for the simulation of high frequency variability in the ESRUN because the instrumental record is of sufficient length to characterize such variability and because many reconstructions obviously underestimate high frequency variability (probably to due temporal resolution limitations of several proxies). On the other hand, it is valuable to estimate low frequency variability using the reconstructions because they are of longer length than the instrumental record and they are from a time period characterized by weaker external forcing.

We chose 15 years as the division between high and low frequency variability because this serves as a natural division between ENSO-like variability (which has a characteristic timescale of ~3-7 years) and slower moving modes of variability such as the Atlantic Multidecadal Oscillation and Pacific Decadal Oscillation which have timescales of multiple decades[6]. Also the 15-year division allowed the first three IMFs from the instrumental record (Fig. S2d) to be included in creation of the ESRUN. The timescales of variability for these first three IMFs indicate that they likely arose from unforced internal dynamics. On the contrary, the 4th instrumental IMF (Fig. S2d) cools from the late 1800s to ~1910 and then warms from ~1910 to ~1940. It is possible that this variability in particular is due to external forcings[7] that have not been completely removed by the Multiple Linear Regression procedure. Because of ambiguity between forced and unforced variability in the instrumental record at this and longer timescales, it is valuable to use additional estimates of low frequency variability provided by the reconstructions.

Figures S7 and S8 show the primary results of the manuscript when the high/low frequency division is assigned to be 35 years which allows the first four IMFs from the instrumental record (Fig. S2d) to be included in the ESRUN. Figures S7 and S8 indicate that the primary results are similar for both cutoff frequencies but that there is slightly less energetic low frequency variability in the ESRUN when the cutoff frequency is 35 years. This may be because some of the low frequency variability in the instrumental record has been mistaken for forced variability and the radiative forcings have been implicitly over-fit to the observed GMT at the 15-35 year timescale.

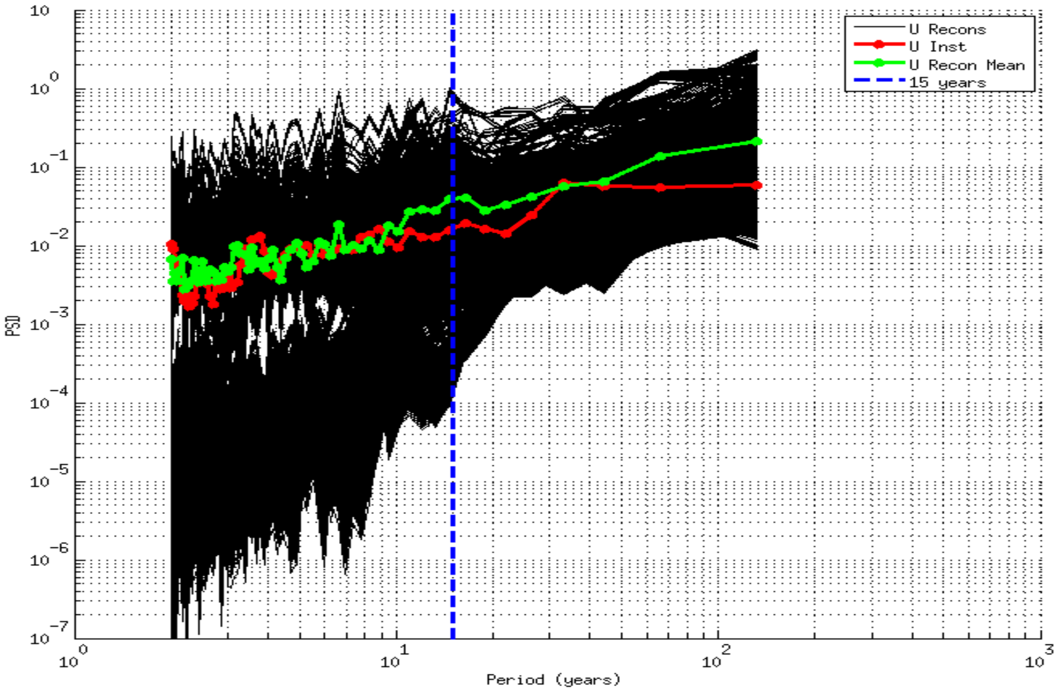

Figure S6 | Power spectrum of unforced GMT from the instrumental record compared to the 15,120 unforced GMT estimates from the reconstructions. The single instrumental power spectrum is shown in red while the individual reconstructed power spectra are shown in black. The mean across all reconstructions is shown in green. The power spectra were calculated using a multi-taper method with adaptive weighing. Only those timescales where instrumental and reconstructed variability overlap are shown. The vertical dashed line delineates where the division is made between high and low frequency variability: the ESRUN uses the instrumental record for high frequency variability (left of the blue dashed line) and uses the reconstructed records for low frequency variability (right of the blue dashed line).

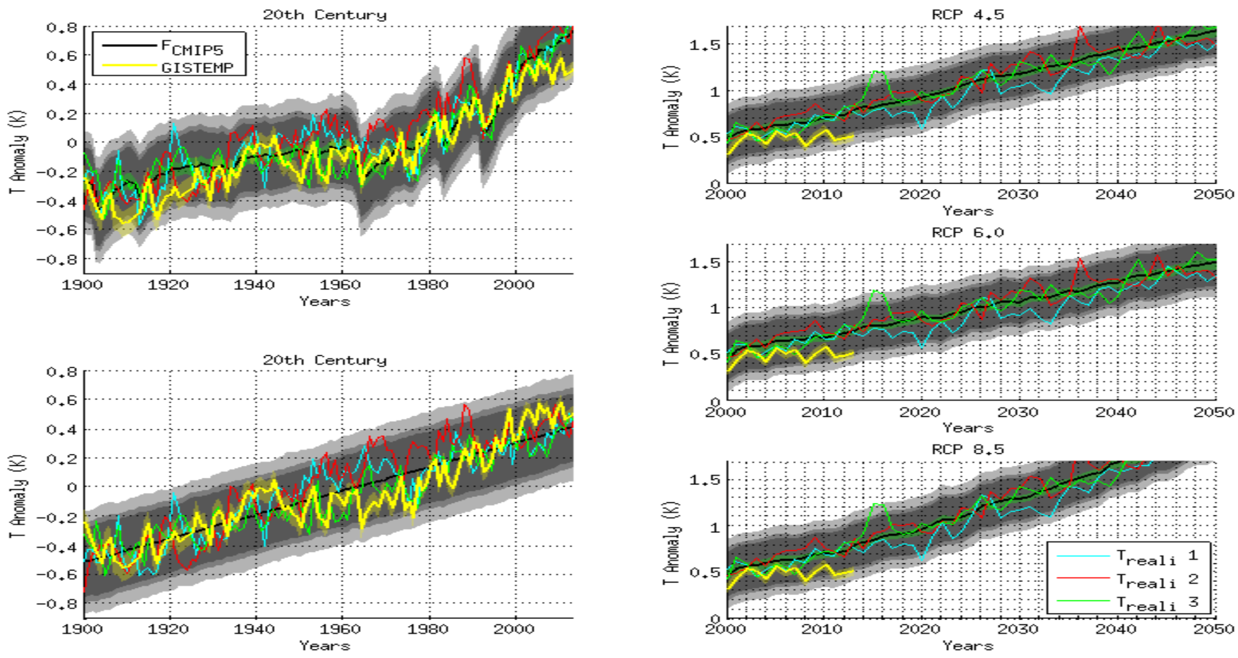

Figure S7 | Same as Fig. 2 but with low frequency variability defined as IMFs with an average wavelength of 35 years or greater.

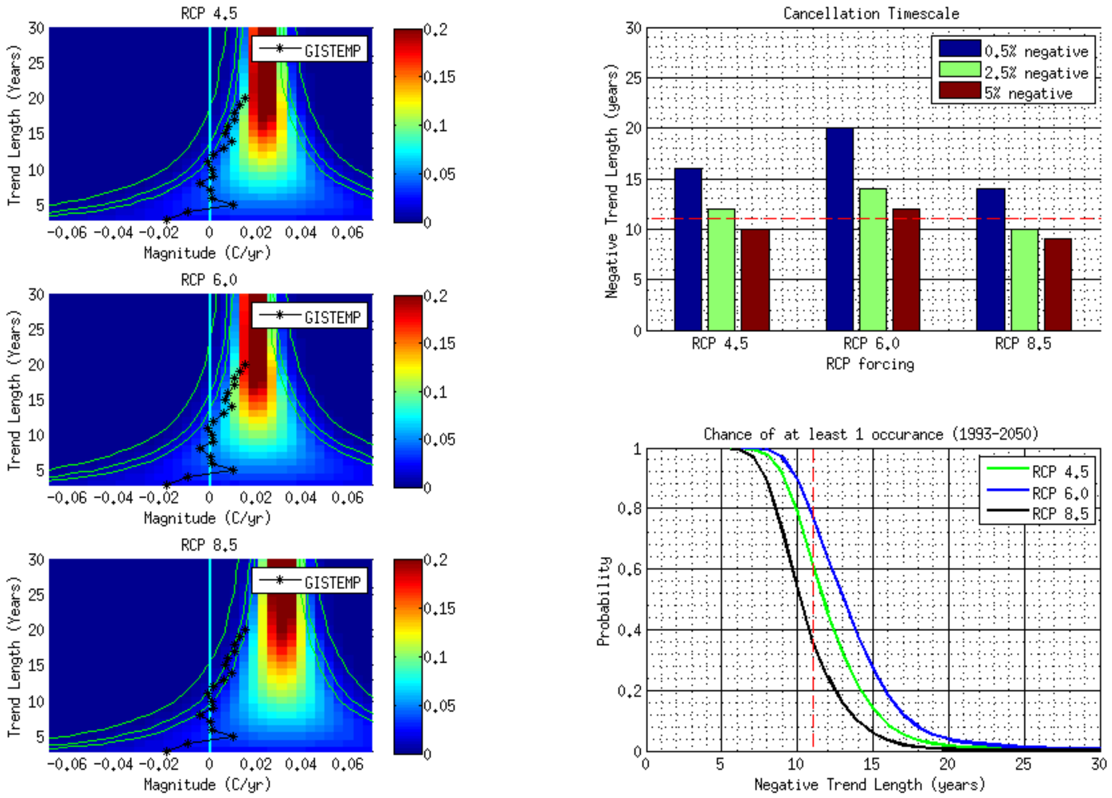

Figure S8 | Same as Fig. 3 but with low frequency variability defined as IMFs with an average wavelength of 35 years or greater.

Sensitivity of Results to AR Order Assignment

Figures S9 and S10 show the main results of the manuscript when Bayesian Information Criterion (BIC)[8] was used at Step 4 (Fig. 4) to assign the autoregressive model order with the best balance between goodness-of-fit and model complexity. In this case, the model order was selected [among AR(1), AR(2),…,AR(10)] that minimized the BIC. Comparison of Figs. S9 and S10 with Figs. 2 and 3 respectively, indicates that the primary results of the manuscript are very similar when BIC is used to assign AR model order and when the model order is pre-assigned to be AR(2).

Figs. S11 and S12 also show very similar results when model order is pre-assigned to be AR(7). This is in contrast to Figs. S13 and S14 where model order was pre-assigned to be AR(1) and there is noticeably less low frequency variability present in the ESRUN.

Overall, these results indicate that AR(1) models are unable to simulate some low frequency variability present in the unforced GMT time series. However, results are very similar when AR(2), AR(7) or AR(X) [where X is assigned from 1 to 10 using BIC] models are used to create the ESRUN. Therefore, it appears that only marginal improvements are achieved by increasing model order past AR(2) and thus we present the AR(2) results in the main text of the manuscript. The ability of AR(2) models to simulate the unforced GMT time series is also illustrated in Figs. S3 and S5.

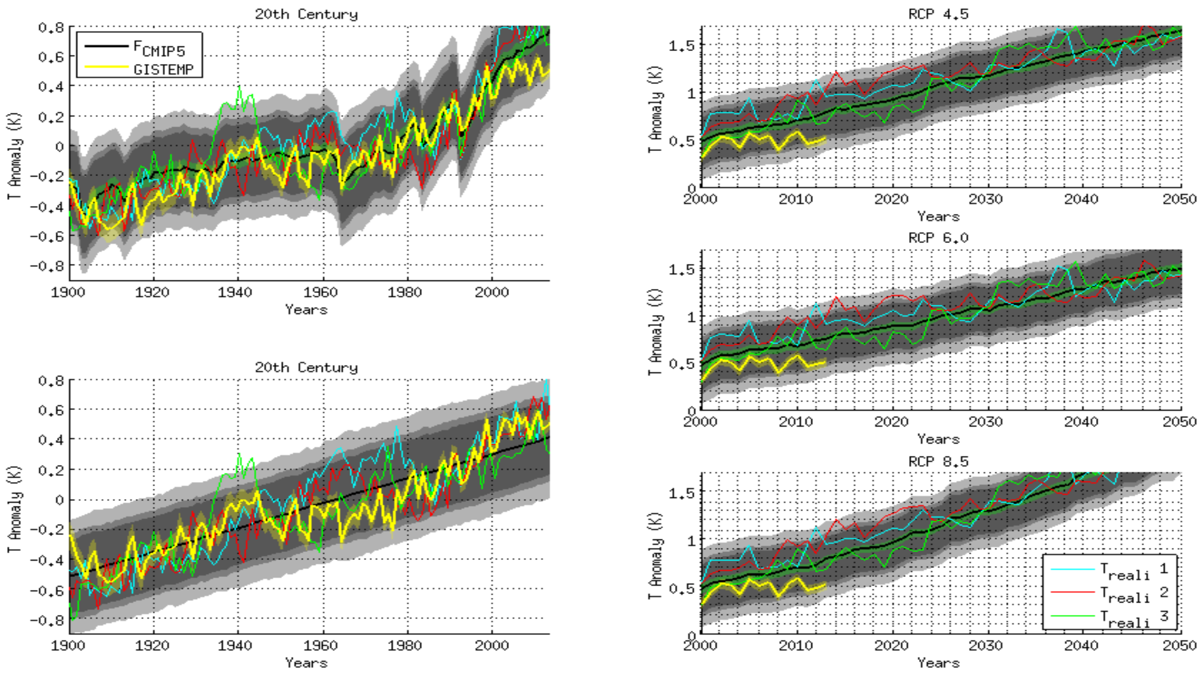

Figure S9 | Same as Fig. 2 but with AR model order assigned using BIC. Possible model orders ranged from AR(1) to AR(10) for each IMF.

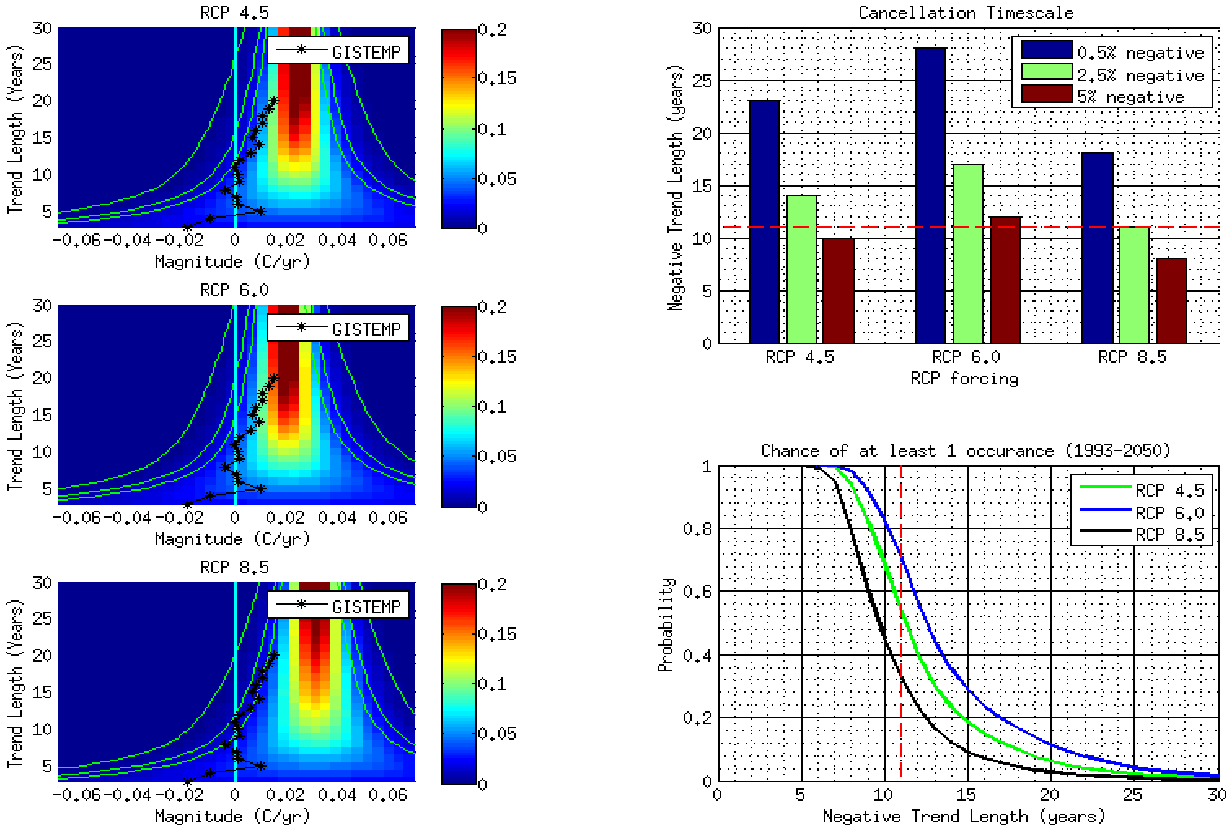

Figure S10 | Same as Fig. 3 but with AR model order assigned using BIC. Possible orders ranged from AR(1) to AR(10) for each IMF.

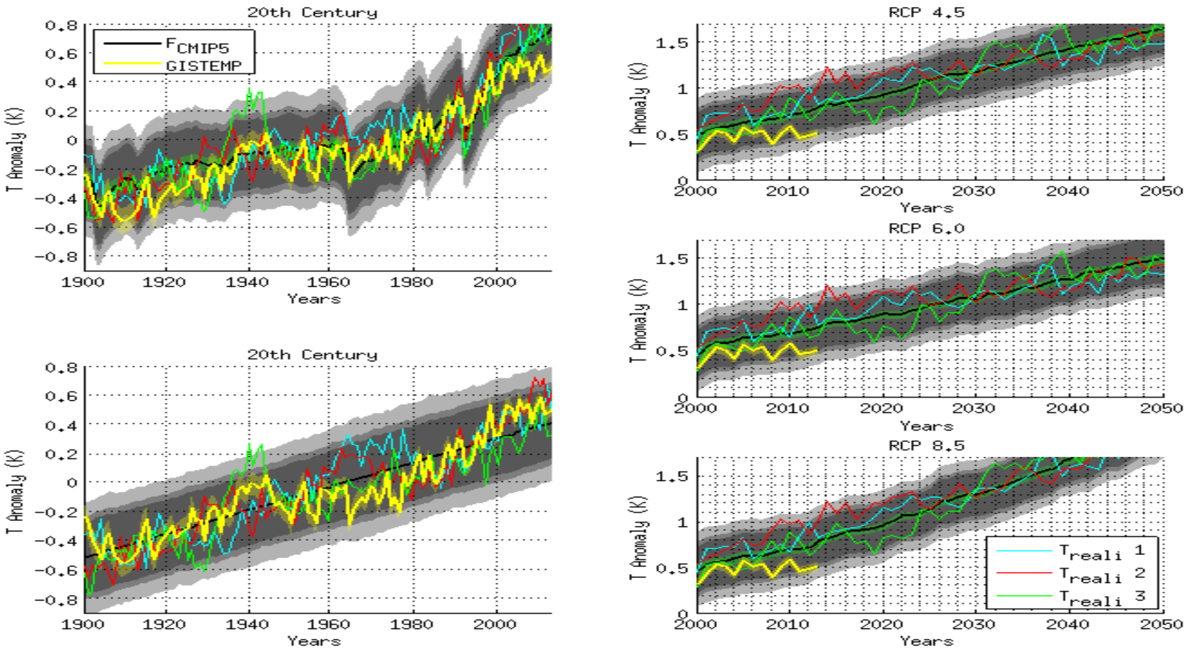

Figure S11 | Same as Fig. 2 but with AR(7) models used to simulate the IMFs

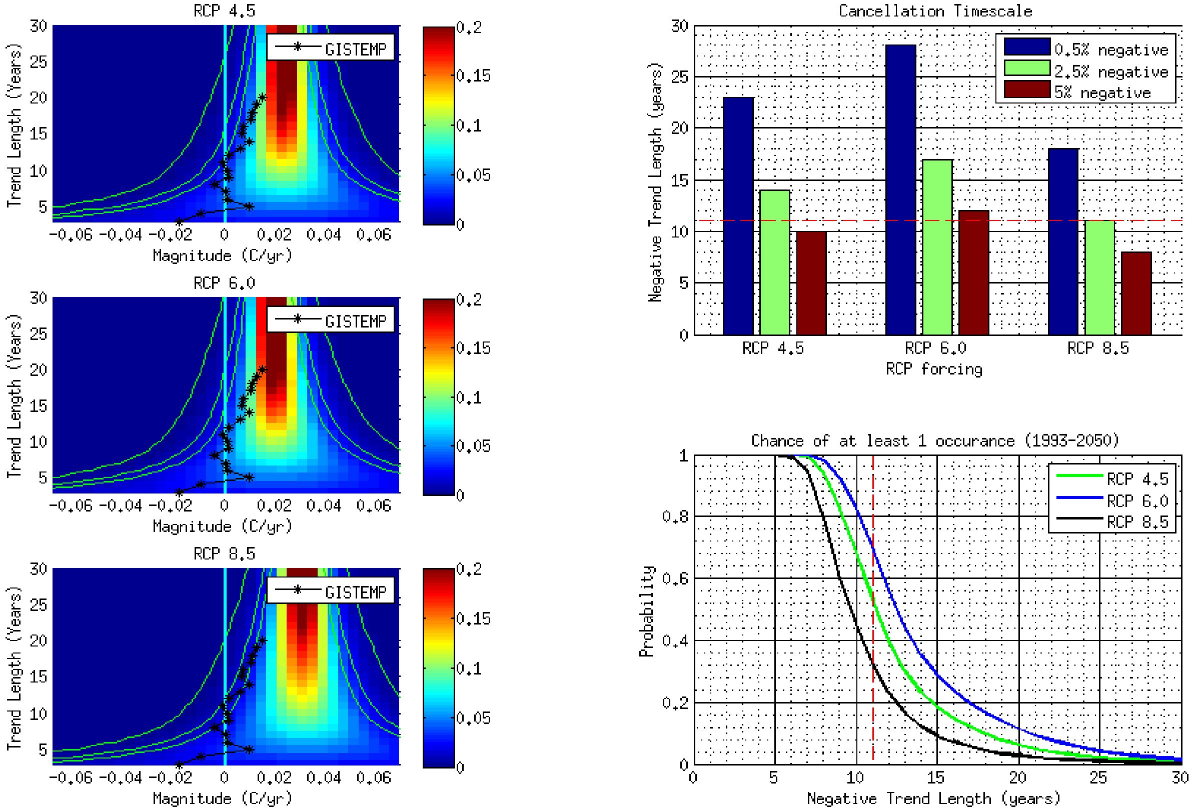

Figure S12 | Same as Fig. 3 but with AR(7) models used to simulate the IMFs

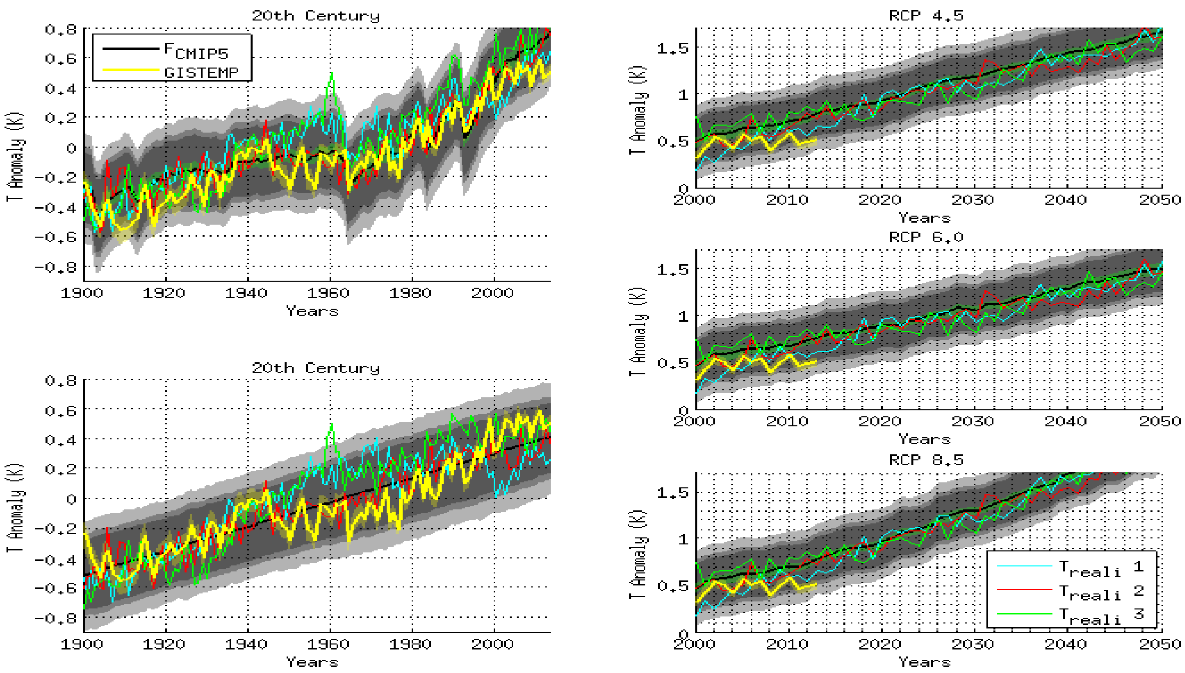

Figure S13 | Same as Fig. 2 but with AR(1) models used to simulate the IMFs

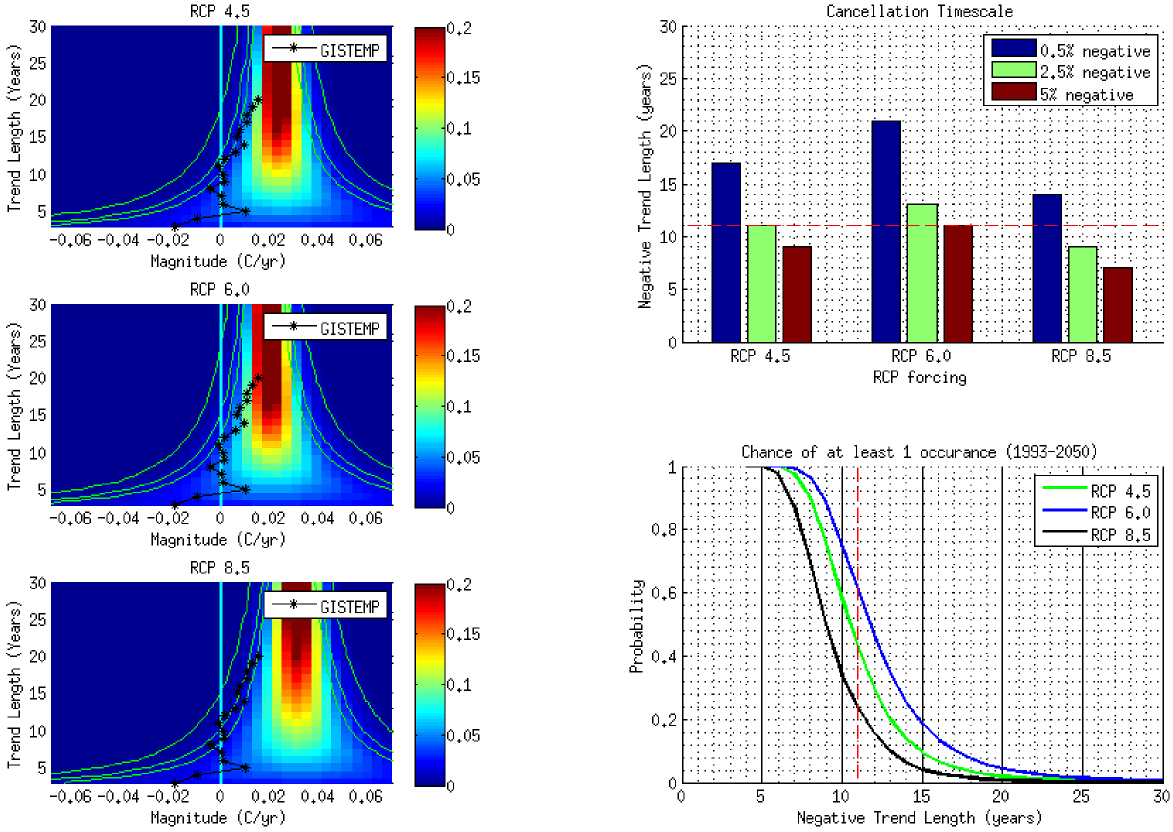

Figure S14 | Same as Fig. 3 but with AR(1) models used to simulate the IMFs

Reconstruction information

The following is information on the surface temperature reconstructions used in this study. The information below comes directly from Wahl, et al. [9]

Ammann and Wahl [10]

TITLE: Northern Hemisphere Average Annual Temperature Reconstruction

DESCRIPTION_SUMMARY: Uses multiple proxy types, input into inverse regression-truncated EOF climate field reconstruction spanning entire globe at incomplete 5x5 deg grid. Only N. Hemisphere average is reported here.

Briffa, et al. [11]

TITLE: Northern Hemisphere Temperature Reconstructions

DESCRIPTION_SUMMARY: Derived from means of 383 maximum latewood density chronologies from the northern Boreal forest.

Crowley [12]

TITLE: Northern Hemisphere Temperature Reconstruction

DESCRIPTION_SUMMARY: Proxies used include tree-rings, pollen, oxygen isotopes, ice core, phenological records, historical records. Modification of reconstruction from Crowley, T.J., and T. S. Lowery. 2000. How Warm was the Medieval Warm Period. Ambio 29:51-54.

D'Arrigo, et al. [13]

A)

TITLE: Northern Hemisphere Tree-Ring-Based Temperature Reconstruction: Standard

DESCRIPTION_SUMMARY: Standard Reconstruction (negative-exponential or straightline curve fits). Tree-ring based reconstruction from 66 high elevation and latitudinal treeline North American and Eurasian sites.

B)

TITLE: Northern Hemisphere Tree-Ring-Based Temperature Reconstruction: Regional Curve Standardization

CITATION: D'Arrigo, R., R. Wilson, and G. Jacoby. 2006. On the long-term context for late twentieth century warming. Journal of Geophysical Research 111:D03103. DOI: 10.1029/2005JD006352.

Huang [14]

TITLE: Integrated Northern Hemisphere Surface Temperature Reconstruction

DESCRIPTION_SUMMARY: Reconstruction based on borehole temperatures, the 20th century meteorological record, and multi-proxy paleoclimatic records.

Jones, et al. [15]

A)

TITLE: Millennial Temperature Reconstructions: Northern Hemisphere

DESCRIPTION_SUMMARY: tree rings, ice cores, corals, and historical documents

B)

TITLE: Millennial Temperature Reconstructions: Southern Hemisphere

DESCRIPTION_SUMMARY: tree rings, ice cores, corals, and historical documents

Mann, et al. [16]

TITLE: Northern Hemisphere Temperatures During the Past Millennium

DESCRIPTION_SUMMARY: Proxies used include tree-rings, ice cores, corals, long historical records, and long instrumental data series. Extension over 1000 AD to 1399 AD of Mann, M.E., R.S. Bradley, and M.K. Hughes. 1998. Global-Scale Temperature Patterns and Climate Forcing Over the Past Six Centuries. Nature 392:779-787.

Mann and Jones [17]

A)

TITLE: 2,000 Year Hemispheric Multi-proxy Temperature Reconstructions: Global

DESCRIPTION_SUMMARY: Tree-rings, historical records, lake sediments, ice cores, fossil shells, and boreholes. Decadally-resolved series.

B)

TITLE: 2,000 Year Hemispheric Multi-proxy Temperature Reconstructions: Northern Hemisphere

DESCRIPTION_SUMMARY: Tree-rings, historical records, lake sediments, ice cores, fossil shells, and boreholes. Decadally-resolved series

C)

TITLE: 2,000 Year Hemispheric Multi-proxy Temperature Reconstructions: Southern Hemisphere

DESCRIPTION_SUMMARY: Tree-rings, historical records, lake sediments, ice cores, fossil shells, and boreholes. Decadally-resolved series

Mann, et al. [18]

B)

TITLE: 2,000 Year Hemispheric and Global Surface Temperature Reconstructions: Northern Hemisphere: Land and Ocean: Composite Plus Scale Method

DESCRIPTION_SUMMARY: Proxies include tree-ring, marine sediment, speleothem, lacustrine, ice core, coral, and historical documentary series. Composite reconstruction formed by averaging all validated reconstruction scenarios for the given reconstruction method and spatial target. Cf. page 13255 of original publication and Supporting Information Figures S5 and S6.

D)

TITLE: 2,000 Year Hemispheric and Global Surface Temperature Reconstructions: Southern Hemisphere: Land and Ocean: Composite Plus Scale Method

CITATION: Mann, M.E., Z. Zhang, M.K. Hughes, R.S. Bradley, S.K. Miller, S. Rutherford, and F. Ni. 2008. Proxy-based reconstructions of hemispheric and global surface temperature variations over the past two millennia. Proceedings of the National Academy of Sciences 105:13252-13257. DOI:10.1073/pnas.0805721105.

F)

TITLE: 2,000 Year Hemispheric and Global Surface Temperature Reconstructions: Global: Land and Ocean: Error-In-Variables Method

DESCRIPTION_SUMMARY: Proxies include tree-ring, marine sediment, speleothem, lacustrine, ice core, coral, and historical documentary series. Error-In-Variables (EIV) based on RegEM algorithm. Composite reconstruction formed by averaging all validated reconstruction scenarios for the given reconstruction method and spatial target. Cf. page 13255 of original publication and Supporting Information Figures S5 and S6.

H)

TITLE: 2,000 Year Hemispheric and Global Surface Temperature Reconstructions: Northern Hemisphere: Land and Ocean: Error-In-Variables Method

DESCRIPTION_SUMMARY: Proxies include tree-ring, marine sediment, speleothem, lacustrine, ice core, coral, and historical documentary series. Error-In-Variables (EIV) based on RegEM algorithm. Composite reconstruction formed by averaging all validated reconstruction scenarios for the given reconstruction method and spatial target. Cf. page 13255 of original publication and Supporting Information Figures S5 and S6.

J)

TITLE: 2,000 Year Hemispheric and Global Surface Temperature Reconstructions: Southern Hemishpere: Land and Ocean: Error-In-Variables Method

DESCRIPTION_SUMMARY: Proxies include tree-ring, marine sediment, speleothem, lacustrine, ice core, coral, and historical documentary series. Error-In-Variables (EIV) based on RegEM algorithm. Composite reconstruction formed by averaging all validated reconstruction scenarios for the given reconstruction method and spatial target. Cf. page 13255 of original publication and Supporting Information Figures S5 and S6.

Moberg, et al. [19]

TITLE: 2,000-Year Northern Hemisphere Temperature Reconstruction

DESCRIPTION_SUMMARY: Reconstruction calculated by combining low-resolution proxies with tree-ring data, using a wavelet transform technique.

References

1 Hansen, J. *et al.* Climate simulations for 1880–2003 with GISS modelE. *Climate Dynamics* **29**, 661-696, doi:10.1007/s00382-007-0255-8 (2007).

2 Huang, N. E. *et al.* The empirical mode decomposition and the Hilbert spectrum for nonlinear and non-stationary time series analysis. *Proceedings of the Royal Society of London. Series A: Mathematical, Physical and Engineering Sciences* **454**, 903-995, doi:10.1098/rspa.1998.0193 (1998).

3 Goosse, H. & Renssen, H. Exciting natural modes of variability by solar and volcanic forcing: idealized and realistic experiments. *Climate Dynamics* **23**, 153-163, doi:10.1007/s00382-004-0424-y (2004).

4 Ottera, O. H., Bentsen, M., Drange, H. & Suo, L. External forcing as a metronome for Atlantic multidecadal variability. *Nature Geosci* **3**, 688-694, (2010).

5 Mann, M. E. *et al.* Global Signatures and Dynamical Origins of the Little Ice Age and Medieval Climate Anomaly. *Science* **326**, 1256-1260, doi:10.1126/science.1177303 (2009).

6 Deser, C., Alexander, M. A., Xie, S.-P. & Phillips, A. S. Sea Surface Temperature Variability: Patterns and Mechanisms. *Annual Review of Marine Science* **2**, 115-143, doi:doi:10.1146/annurev-marine-120408-151453 (2010).

7 Stott, P. A. *et al.* External Control of 20th Century Temperature by Natural and Anthropogenic Forcings. *Science* **290**, 2133-2137, doi:10.1126/science.290.5499.2133 (2000).

8 Box, G. E. P. G. M. J. G. C. R. *Time Series Analysis: Forecasting and Control*. 3 edn, (Prentice Hall, 1994).

9 Wahl, E. R. *et al.* An archive of high-resolution temperature reconstructions over the past 2+ millennia. *Geochemistry, Geophysics, Geosystems* **11**, Q01001, doi:10.1029/2009GC002817 (2010).

10 Ammann, C. & Wahl, E. The importance of the geophysical context in statistical evaluations of climate reconstruction procedures. *Climatic Change* **85**, 71-88, doi:10.1007/s10584-007-9276-x (2007).

11 Briffa, K. R., Jones, P. D., Schweingruber, F. H. & Osborn, T. J. Influence of volcanic eruptions on Northern Hemisphere summer temperature over the past 600 years. *Nature* **393**, 450-455, (1998).

12 Crowley, T. J. Causes of Climate Change Over the Past 1000 Years. *Science* **289**, 270-277, doi:10.1126/science.289.5477.270 (2000).

13 D'Arrigo, R., Wilson, R. & Jacoby, G. On the long-term context for late twentieth century warming. *Journal of Geophysical Research: Atmospheres* **111**, D03103, doi:10.1029/2005JD006352 (2006).

14 Huang, S. Merging information from different resources for new insights into climate change in the past and future. *Geophysical Research Letters* **31**, L13205, doi:10.1029/2004GL019781 (2004).

15 Jones, P. D., Briffa, K. R., Barnett, T. P. & Tett, S. F. B. High-resolution palaeoclimatic records for the last millennium: interpretation, integration and comparison with General Circulation Model control-run temperatures. *The Holocene* **8**, 455-471, doi:10.1191/095968398667194956 (1998).

16 Mann, M. E., Bradley, R. S. & Hughes, M. K. Northern hemisphere temperatures during the past millennium: Inferences, uncertainties, and limitations. *Geophysical Research Letters* **26**, 759-762, doi:10.1029/1999GL900070 (1999).

17 Mann, M. E. & Jones, P. D. Global surface temperatures over the past two millennia. *Geophysical Research Letters* **30**, 1820, doi:10.1029/2003GL017814 (2003).

18 Mann, M. E. *et al.* Proxy-based reconstructions of hemispheric and global surface temperature variations over the past two millennia. *Proceedings of the National Academy of Sciences*, doi:10.1073/pnas.0805721105 (2008).

19 Moberg, A., Sonechkin, D. M., Holmgren, K., Datsenko, N. M. & Karlen, W. Highly variable Northern Hemisphere temperatures reconstructed from low- and high-resolution proxy data. *Nature* **433**, 613-617, (2005).
